# Development of Posterior Hypothalamic Neurons Enlightens a Switch in the Prosencephalic Basic Plan

**DOI:** 10.1371/journal.pone.0028574

**Published:** 2011-12-16

**Authors:** Sophie Croizier, Clotilde Amiot, Xiaoping Chen, Françoise Presse, Jean-Louis Nahon, Jane Y. Wu, Dominique Fellmann, Pierre-Yves Risold

**Affiliations:** 1 EA3922, Faculté de Médecine et de Pharmacie, Université de Franche-Comté, Besançon, France; 2 IFR133, Université de Franche-Comté, Besançon, France; 3 Department of Neurology, School of Medicine, Northwestern University, Chicago, Illinois, United States of America; 4 UMR 6097 CNRS, Institut de Pharmacologie Moléculaire et Cellulaire, Université Nice-Sophia Antipolis, Valbonne, France; Instituto de Medicina Molecular, Portugal

## Abstract

In rats and mice, ascending and descending axons from neurons producing melanin-concentrating hormone (MCH) reach the cerebral cortex and spinal cord. However, these ascending and descending projections originate from distinct sub-populations expressing or not “Cocaine-and-Amphetamine-Regulated-Transcript” (CART) peptide. Using a BrdU approach, MCH cell bodies are among the very first generated in the hypothalamus, within a longitudinal cell cord made of earliest delaminating neuroblasts in the diencephalon and extending from the chiasmatic region to the ventral midbrain. This region also specifically expresses the regulatory genes Sonic hedgehog (Shh) and Nkx2.2. First MCH axons run through the *tractus postopticus* (*tpoc*) which gathers pioneer axons from the cell cord and courses parallel to the Shh/Nkx2.2 expression domain. Subsequently generated MCH neurons and ascending MCH axons differentiate while neurogenesis and mantle layer differentiation are generalized in the prosencephalon, including telencephalon. Ascending MCH axons follow dopaminergic axons of the mesotelencephalic tract, both being an initial component of the medial forebrain bundle (mfb). Netrin1 and Slit2 proteins that are involved in the establishment of the *tpoc* and mfb, respectively attract or repulse MCH axons.

We conclude that first generated MCH neurons develop in a diencephalic segment of a longitudinal Shh/Nkx2.2 domain. This region can be seen as a prosencephalic segment of a medial neurogenic column extending from the chiasmatic region through the ventral neural tube. However, as the telencephalon expends, it exerts a trophic action and the mfb expands, inducing a switch in the longitudinal axial organization of the prosencephalon.

## Introduction

Neurons producing melanin-concentrating hormone form a very conspicuous cell population in the dorsal and lateral hypothalamus [1]. They are involved in sleep/wake cycle, and project throughout the central nervous system [1], [2], [3], as other diffusely projecting hypocretin (Hcrt)-, histamin- or serotonin-containing cell groups [4], [5], [6], [7]. MCH neurons are also involved in reward and reinforcement responses associated with feeding [8], [9], [10]. Dopamine regulates their electrical activity through multiple pre- and post-synaptic mechanisms [11]. In addition, the MCH peptide modulates the dopaminergic mesotelencephalic system in the ventral tegmental area and accumbens nucleus [12]. It acts in these structures at least partly in concert with CART (‘cocaine and amphetamine related transcript’ peptide) and GABA [12], [13].

To date, the specific morphofunctional organization of the whole MCH population is not clearly understood [9], [14]. At least two, but maybe more, MCH sub-populations exist [15], [16]. These sub-populations seem not associated with particular functions but their differentiation results of unknown developmental events [2], [17], [18], [19].

In the rat embryo, MCH neurons are localized in a very restricted region of the diencephalic wall [17], despite the fact that in adult animals MCH neurons are observed in seven hypothalamic structures and the adjacent *zona incerta* of the ventral (or pre-) thalamus [1], [20], [21]. A great deal has been recently learned on mechanisms governing the development of the diencephalon, and concerning the expression patterns of developmental regulatory genes [22].

However, gene expression patterns are not sufficient to understand the adult MCH morphofunctional organization. The stage at which a particular MCH neuron is generated influences the anatomical location of its soma, its projection pattern but also the co-expression of CART and NK3. In rat, most MCH neurons that send projections in the spinal cord are generated before E12, while more than 80% of MCH cells that project into the cerebral cortex are produced at or after E12. CART and NK3 are expressed in neurons of this second sub-population [15], [16], [17].

Therefore, the present study had two objectives: first, to characterize the very well circumscribed MCH embryonic area with regard to early neurogenesis in the diencephalon and expression patterns of regulatory genes. Second, to study the timely development of descending and ascending MCH projections correlated to the development of pioneering axon tracts. Because of its early genesis and phenotype differentiation, the MCH system proves to be a key model enlightening early mechanisms of the prosencephalic development.

## Results

### Differentiation of MCH neurons in the primary diencephalic mantle layer

In the rat, the peak of birth of MCH neurons that project into the spinal cord is at E11, but the MCH phenotype differentiates only two to three days later (E13/14) [17]. Therefore, as a first step to understand the succession of events involved in the differentiation of MCH divergent projection patterns, a sequential BrdU analysis was undertaken. The main objective of this experiment was to compare the distribution of early generated MCH neurons to the general pattern of neurogenesis in the hypothalamus. The second objective was to verify that the first perikarya labeled for MCH are also generated first.

**Figure 1:**
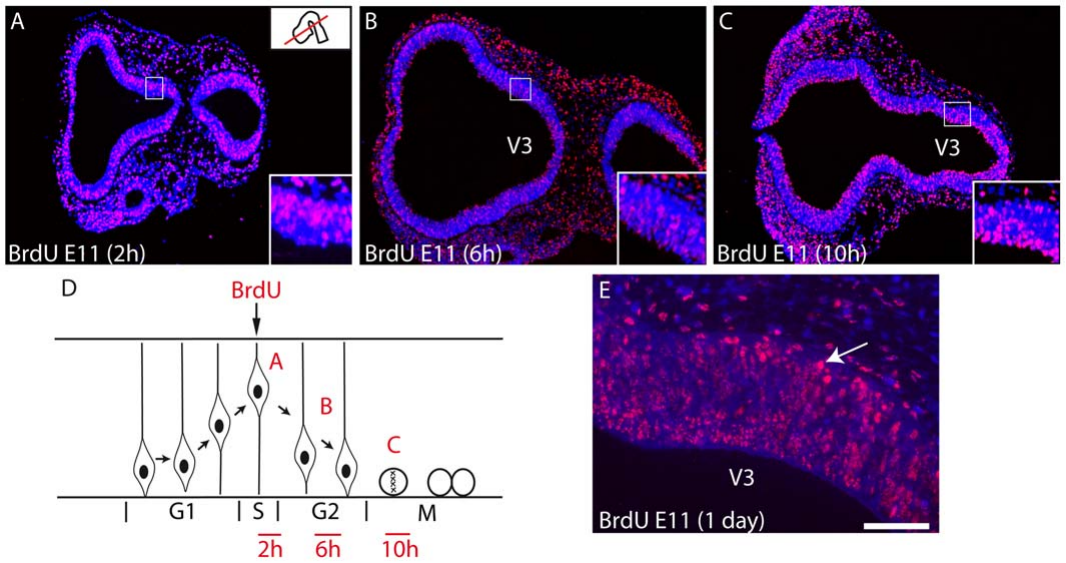
BrdU labeling 2, 6, 10 or 24 h after injection at E11. Photomicrographs illustrating BrdU labeled nuclei on horizontal sections of the prosencephalon after E11 BrdU injections in the rat. Sections are counterstained with DAPI. Embryos were sacrificed 2, 6 or 10 hours after injection. The distribution of BrdU-labeled nuclei highlights interkinetic movement of these nuclei within the neuroepithelium. (A) Two hours after the BrdU injection, labeled nuclei are external in the neuroepithelium, suggesting that cells are in the S phase. (B) Six hours after injection, labeled nuclei are observed through the thickness of the neuroepithelium, suggesting that these nuclei belong to cells in G2 phase. (C) Finally, ten hours after injection, the ponctiform and intense labeling close to the ventricular surface suggests that mitotic neurons are labeled. (D) Scheme representing the interkinetic movement of nuclei within the neuroepithelium and summarizing the distribution of the BrdU signal as illustrated in A–C. (E) 24 hours after injection, some intensely labeled nuclei are outside of neuroepithelium and invade the dawning mantle layer of dorsal posterior hypothalamus. Scale bar: A–C = 500 µm; E = 100 µm. V3: third ventricle.

**Figure 2:**
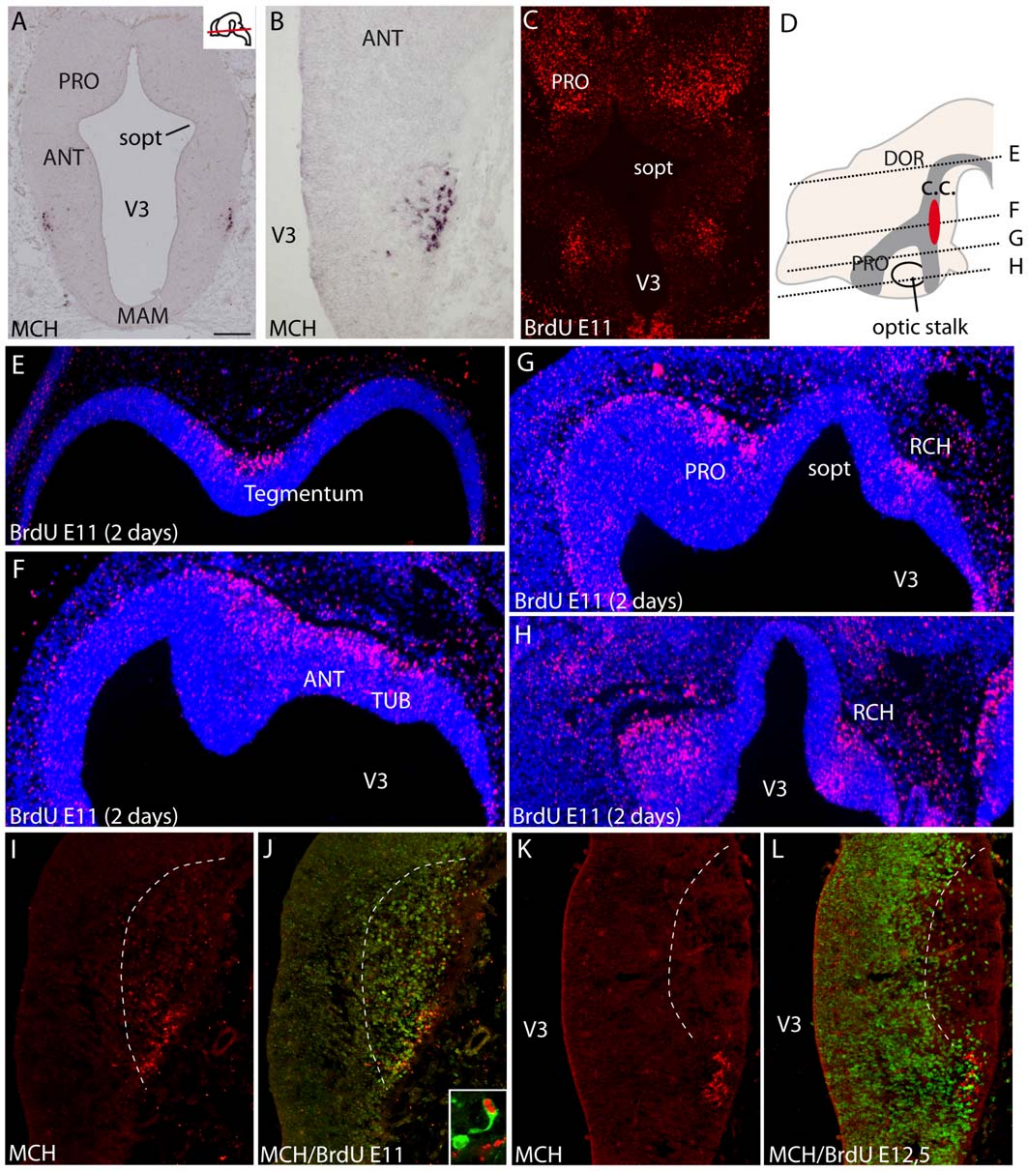
Genesis of MCH neurons. (A, B) Distribution of preproMCH *in situ* signal on horizontal sections of an E14 rat embryo. MCH neurons are in a restricted region of the dorsal hypothalamic mantle layer. (C) Distribution of BrdU-labeled nuclei on an E13 rat brain section passing through the ventral hypothalamus after a single BrdU injection at E11. (D–H) Photomicrographs showing BrdU immunohistochemistry on E13 rat horizontal sections counterstained with DAPI, and after BrdU injection at E11; in D, the distribution of BrdU-labeled nuclei (dark grey area) is schematized on a sagittal drawing of the embryonic brain; the red area represent MCH neurons. Sections are arranged from dorsal (E) to ventral (H). BrdU-labeled nuclei are located within a longitudinal region extending from the chiasmatic region to the ventral midbrain, and extending in the preoptic region and ventral telencephalon. (I–L) Distribution of MCH (red) and BrdU-labeled nuclei (green) after multiple injections at E11 (I, J) or E12.5 (K, L). Note the complementary BrdU patterns. Frame in J is a confocal picture of a double labeled MCH/BrdU neuron. For esthetic purpose BrdU is in red and MCH in green. Scale bars: A–C = 500 µm; B, I–L = 250 µm; E–H = 400 µm. ANT: anterior level, hypothalamus; c.c.: ‘cell cord’; MAM: mammillary level, hypothalamus; PRO: preoptic level, hypothalamus; RCH: retrochiasmatic area; sopt: optic sulcus; TUB: tuberal level, hypothalamus; V3: third ventricle.

Rat E11 pregnant females were injected with BrdU, and the distribution of BrdU labeled nuclei was analyzed on series of cryostat or paraffin embedded sections of embryos taken 2, 6, 10, 24 (E12), 48 (E13), 72 (E14) hours after injection. The sequential analysis of BrdU pattern only hours (2–10 h) post-injections enlightened the interkinetic movement of nuclei within the neuroepithelium (Figure 1A–D). Labeled nuclei outside the neuroepithelium, evocating newborn neurons in the dawning mantle layer, were observed on the E12 material (24 hours after BrdU injection) in the presumptive dorsal hypothalamic region, just dorsal to the optic stalk (Figure 1E). At E13 and E14, the distribution pattern of E11 BrdU nuclei was fully reminiscent of the development of the primary prosencephalic mantle layer described by Keyser [23], [24]. At the same stages, a cluster of MCH cells is labeled in the mantle layer using in situ hybridization or immunohistochemistry (Figure 2A, B). Intensely BrdU-labeled nuclei were located within a longitudinal region extending from the chiasmatic region to the ventral midbrain. At dorsal hypothalamic levels, intensely labeled BrdU nuclei were also observed in a region extending in the anterior hypothalamus and ventral telencephalic vesicle (Figure 2C–H). In our material, the preoptic region was also labeled (Figure 2C–D, G).

At E14, using double immunohistochemistry, neurons contained both BrdU and MCH labeling (Figure 2I,J). However, these neurons were few in number. To increase the odds of detecting MCH/BrdU cells, pregnant females received four injections of BrdU every two hours from E11 to E11.5. Following this procedure, 42.8% of MCH neurons displayed an intense BrdU nucleus at E14 (Table 1). MCH neurons containing an incompletely or weakly labeled nucleus were not taken into consideration.

**Table 1:** Number of MCH and MCH/BrdU neurons after single or multiple BrdU injections at E11 or at E12.5.

	BrdU injection	Number of MCH neurons	Number of MCH/BrdU-positive neurons	% MCH/BrdU vs MCH
Single BrdU injection	E11	362	ND	ND
	E12.5	388	ND	ND
Multiple BrdU injection	E11 to E11.5	271	116	42.8
	E12.5 to E13	263	41	15.5

To complete this experiment, E12.5 pregnant rat received a BrdU injection. E12.5 corresponds to the peak of genesis of cortically projecting MCH neurons. Embryos were taken 36 h later (at E14). The distribution of intensely labeled nuclei within the mantle layer of the hypothalamus was far broader than in the E11 injected material. Interestingly, both distribution patterns were complementary (Figure 2I–L). In the posterior hypothalamus, E12.5 labeled nuclei were medially located compared to E11 labeled neuron. These patterns were even clearer in the retrochiasmatic area. After multiple E12.5 BrdU injections, 15.5% of MCH cells contained an intensely BrdU-labeled nucleus (Table 1). This observation suggests that few MCH neurons generated at E12.5 have differentiated the MCH phenotype at E14 in the rat embryo. It is to note here that multiple BrdU injections had an effect on MCH differentiation as far less MCH neurons were counted in material with multiple injections compared to singly injected material (Table 1). BrdU is known to increase the cell cycle duration, which may in part explain this difference [25]. Nevertheless, the present result clearly indicated that MCH cell bodies are among the first generated neurons, and are within the cell cord described by Keyser in the hypothalamus [23], [24].

### Regulatory gene expression and embryonic MCH expression patterns

**Figure 3:**
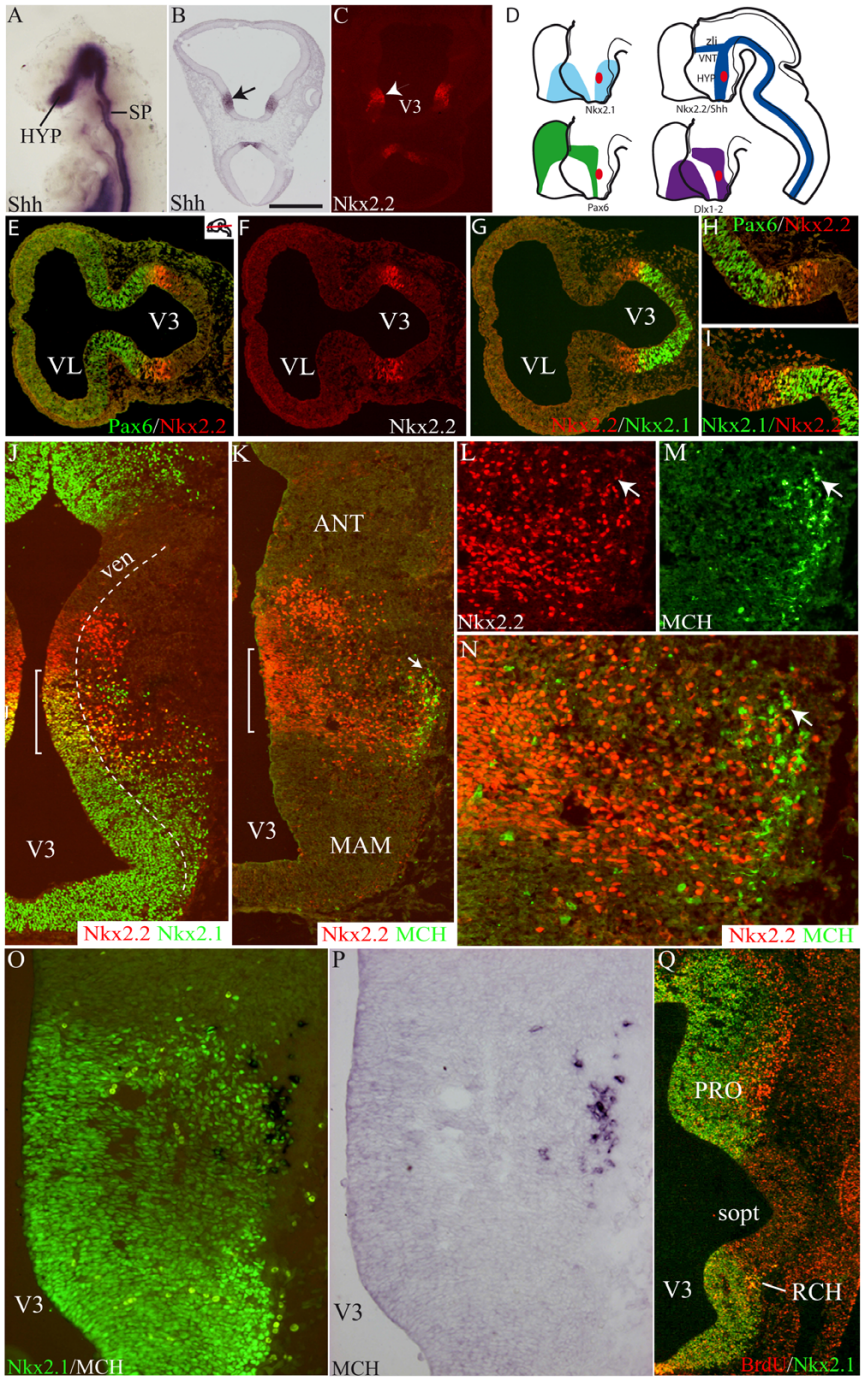
Differentiation of the MCH phenotype and regulatory gene expression patterns. (A) Whole mount Shh *in situ* hybridization of an E9 mouse embryo. (B, C) Shh (*in situ* hybridization – arrow in B) and Nkx2.2 (immunofluorescence – arrowhead in C) on two E10 rat horizontal sections passing through the dorsal hypothalamus. The distribution of both genes is very similar in the diencephalon. (D) Diagrams summarizing the expression patterns of five regulatory genes and MCH (red area) in the prosencephalon. See text for additional details and Shimogori et al. (2010). (E–I) Using double immunofluorescence, distributions of Pax6, Nkx2.1 and Nkx2.2 on two adjacent horizontal sections of an E10 rat embryo. Pax6 and Nkx2.1 expression domains are contiguous. Both encroached upon the Nkx2.2 labeled tissue. (J–N) Photomicrographs to illustrate the immunohistochemical co-distribution of MCH, Nkx2.1 and Nkx2.2 at a low (J, K) or high (L–N) magnifications of a E14 rat embryo. MCH cell bodies are very lateral in the mantle layer (arrow in K) but they are restricted to the Nkx2.2/Nkx2.1 co-expressing region (brackets on J and K) in the dorsal and posterior hypothalamic region. Arrow in L–N points to a MCH/Nkx2.2 perikarya. (O–P) Co-distribution of MCH (*in situ* hybridization) and Nkx2.1 (immunofluorescence); most MCH neurons have a Nkx2.1 labeled nucleus. (Q) Double immunofluorescence labeling for BrdU (red) and Nkx2.1 (green) at E13. Both signals are intense in the preotic and retrochiamatic region. Scale bar: A, H, I, L, M, O, P = 250 µm; B, C = 300 µm; E–G, J, K, Q = 500 µm. ANT: anterior level, hypothalamus; HYP: hypothalamus; MAM: mammillary level, hypothalamus; PRO: preoptic level, hypothalamus; RCH: retrochiasmatic area; sopt: optic sulcus; SP: spinal cord; V3: third ventricle; ven: ventricular layer; VNT: ventral thalamus; *zli*: *zona limitans intrathalamica*.

In a recently published genomic atlas of mouse hypothalamic development, Hcrt-containing neurons (co-localized with MCH cells) and CART neurons (corresponding to MCH cortically projecting cells), derivate from a specific segment of the intrahypothalamica diagonal (ID) described in the hypothalamic anlage [22] (See also [26] for gene expression patterns in the hypothalamus). In the mouse embryo, ID extends from the chiasmatic region to the ventral mesencephalon and is characterized by the expression of Shh. Nkx2.2 expression follows a similar pattern which has been abundantly documented [27], [28] (Figure 3A–C). Furthermore, it was also recently reported that differentiation of MCH and Hcrt phenotypes are Shh dependent [29].

Multiple immunohistochemical labeling combined with *in situ* hybridization were performed to compare the embryonic expression of the MCH peptide or preproMCH (pMCH) mRNA with that of Nkx2.1, Nkx2.2, Dlx1-2, Lhx9 and Pax6. Most meaningful results are shown in Figure 3. We observed that the early MCH-containing region (from E13 through E15) is characterized by the expression in the neuroepithelium of Nkx2.1/Nkx2.2, adjacent to Pax6/Nkx2.2 territories (Figure 3E–N). This region is particularly narrow before differentiation of the mantle layer, but it enlarges while MCH expression increases.

In the mantle layer, neuroblasts expressed both Nkx genes. An expression of Lhx9 and Dlx1-2 was also found in the same region. Lhx6 was not investigated in this study. Nkx2.1 was observed in many MCH neurons if not all (Figure 3O,P). Few MCH cell bodies were also labeled for Dlx1-2 (data not illustrated) or Nkx2.2. None expressed Lhx9 (data not illustrated). The distribution patterns of transcription factors together with MCH are schematized in Figure 3D.

Using double immunohistochemistry, we observed a good correlation in the distribution of E11 BrdU labeled nuclei and Nkx2.1 in the preoptic region (Figure 3Q). In the retrochiasmatic region, BrdU labeled nuclei were restricted to the rostral edge of the Nkx2.1 labeled region, corresponding to the Nkx2.1/Nkx2.2 co-expressing territory. Therefore, a partial correlation exists between transcription factors expression patterns and the early differentiation of the mantle layer, at least in the presumptive preoptic and retrochiasmatic regions.

After E12.5 BrdU injections, the distribution of labeled nuclei was not regionalized in the hypothalamus and did not correspond to distribution patterns of transcription factors.

**Figure 4:**
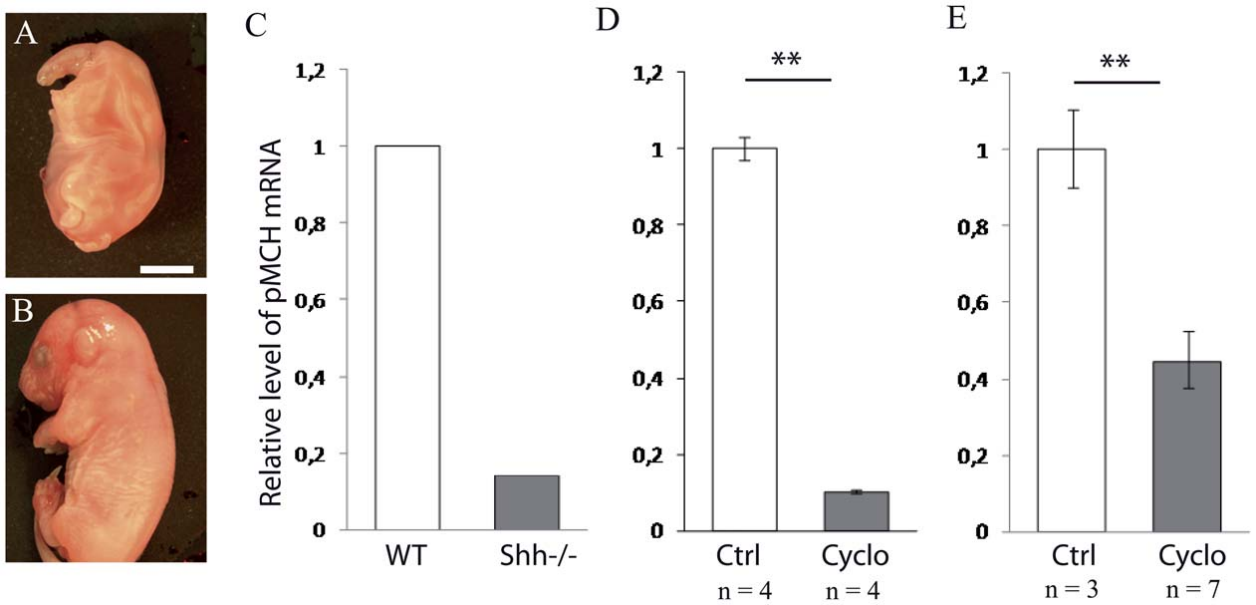
MCH expression is Shh dependent. (A, B) Photomicrographs to illustrate the aspect of a E17 Shh−/− embryo compared to a E17 wild type embryo. The mutant embryo is not viable due to multiple malformations in particular of the head. Scale bar: A = 1,5 mm; B = 4 mm. (C) The relative level of pMCH mRNA was assessed by quantitative real-time PCR in one Shh+/+ and one Shh −/− E13 embryos. pMCH mRNA level was 85% lower in the mutant. (D) The pMCH mRNA level was also decreased by 90% in E13 embryos two days after injection of the Shh pathway inhibitor cyclopamine (cyclo) to the pregnant mice (injection at E11), as compared to control (injection of DMSO, ctrl). (E) pMCH mRNA level is decreased by 55% in E11 half whole brains cultured after two days *in vitro* in presence of cyclopamine. Data indicate mean ± SD, **: p≤0.01, Kruskal Wallis's test.

Finally, an experimental confirmation that the MCH differentiation is Shh dependent was obtained: E11 and E12 Shh^−^/_−_ embryonic mouse brains showed no sign of preproMCH (pMCH) or peptide using *in situ* hybridization or immunohistochemistry, but it was difficult to clearly identify specific structures in the brain of mutant mice as both the head and brain were dramatically different from that of wild type embryos (Figure 4A–B). After RNA extraction from the whole head of a mutant mouse, pMCH mRNA expression was found to be 85% lower compared to a wild type (Figure 4C). Injection of the Shh pathway inhibitor cyclopamine in pregnant mice at E11 resulted two days later in a 90% decrease in the level of pMCH mRNA compared to age matched control embryos (Figure 4D). Finally, mouse E11 half brains incubated with cyclopamine contained significantly less pMCH mRNA than control halves (Figure 4E). All these results indicate that Shh disruption impedes pMCH mRNA expression, reflecting a Shh dependent differentiation of the MCH phenotype.

### First MCH projections follow diencephalic pioneer tracts

**Figure 5:**
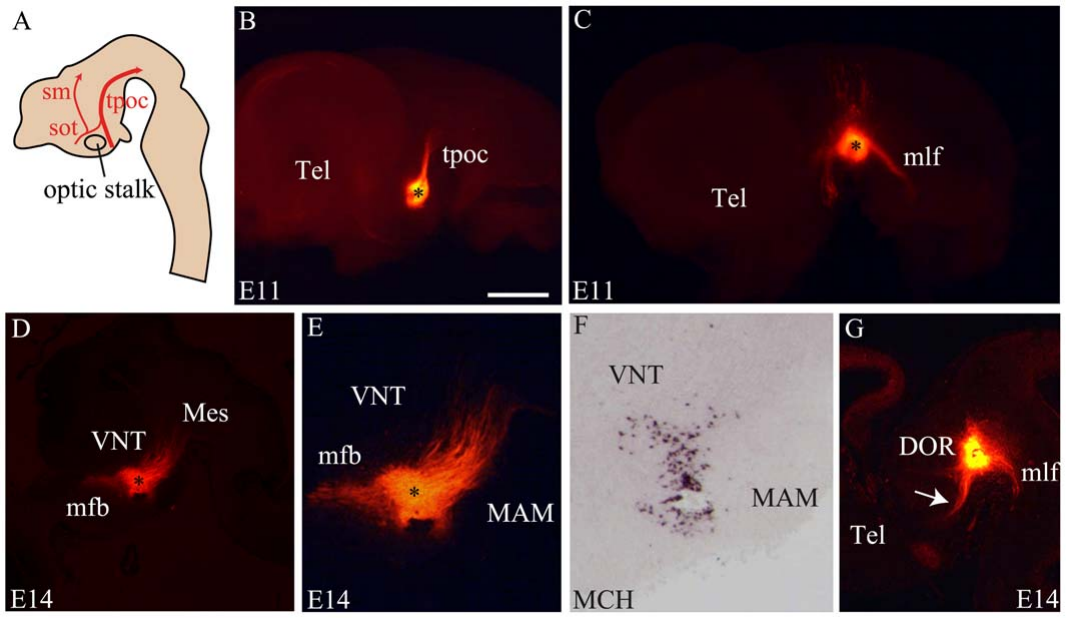
DiI tract tracing from the caudal and dorsal hypothalamus at E11 and E14. (A) Schematic representation of an embryonic brain sagittal section summarizing the position and direction of the *tractus postopticus* (*tpoc* – large red arrow), sot (supraoptic tractus), *sm* (*stria medullaris*). (B) A DiI crystal in the caudal hypothalamus of an E11 mouse labels only the *tpoc*. (C) Control crystal deposited in the ventral mesencephalon of a E11 mouse embryo. The medial longitudinal fascicle (*mlf*), the *tpoc* and dorsally directed fibers in the tegmentum are labeled. (D–F) A DiI crystal deposited within the MCH region (J – *in situ* hybridization for MCH on the same sagittal section) labels caudally but also rostrally directed axons. (G) Control crystals deposited in SN/VTA labeled several tractus as *mlf*, *tpoc*, *mfb* (arrow) or the fasciculus retroflexus. Scale bar: B, C, D, G = 1 mm; E, F = 500 µm. DOR: dorsal thalamus; MAM: mammillary level, hypothalamus; Mes: mesencephalon; Tel: telencephalon; V3: third ventricle; VNT: ventral thalamus.

Pioneer tracts were well described in the mouse prosencephalon ([30] illustrated in Figure 5A). Their differentiation is coincident with neurogenesis and they run longitudinally in the lateral mantle layer of the diencephalon. The *tractus postopticus* (*tpoc*) develops first. This axon bundle runs parallel to the Shh/Nkx2.2 containing domain, from the retrochiasmatic region to the ventral mesencephalon [27]. The *tpoc* was clearly highlighted in E11 mouse embryo after a DiI crystal deposit into the posterior hypothalamic region, as done by Mastick and Easter [30] (Figure 5B). This is the sole tracts detected from the caudal hypothalamus by the lipophilic tracer at this stage. By contrast, a control DiI crystal in the ventral mesencephalon (Figure 5C) labels several rostrally, dorsally and caudally (mlf) directed pathways.

DiI crystals implanted in the MCH-expressing region of E14 mouse embryos labeled caudally directed axons, but also a thick bundle of rostrally directed fibers forming the medial forebrain bundle (mfb) (Figure 5D–F). DiI in the ventral mesencephalon at the same stage labeled axons reaching the posterior hypothalamic region in the mfb (Figure 5G).

**Figure 6:**
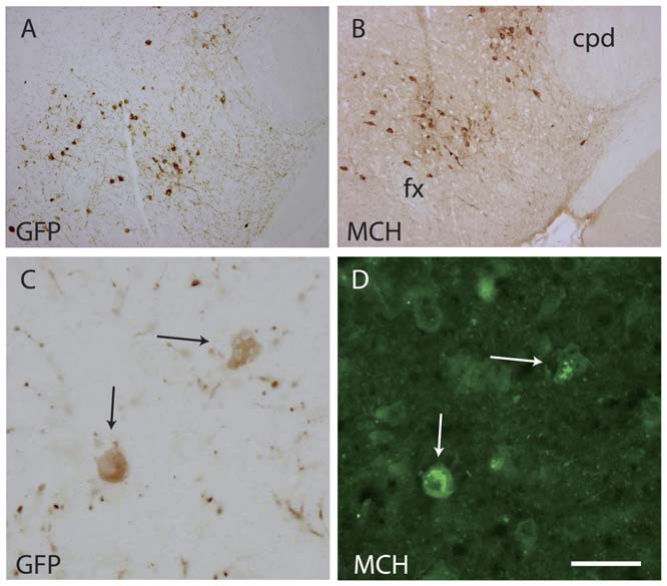
GFP and MCH are expressed by the same cells in MCH-GFP mice. (A, B) Photomicrographs of two adjacent sections passing through the caudal lateral hypothalamus of an adult mouse brain and labeled by the GFP- or MCH-AS using the standard peroxidase anti-peroxydase procedure. Both AS labeled neurons showing similar distribution patterns in the perifornical region (fx: fornix) and adjacent to the cerebral peduncle (cpd). (C, D): A double immunohistochemical procedure (peroxidase – GFP, immunofluresecnce - MCH), both AS revealed the same neurons. Scale bar: A, B = 500 µm; C, D = 50 µm.

The development of MCH perikarya and axons with regard to these tracts was analyzed using a MCH-GFP mouse line. Distribution patterns of perikarya labeled by MCH or GFP antibodies were identical in the hypothalamic anlage. However, GFP detection was more sensitive: axons labeled by the GFP-AS were very abundant while only few could be seen with the MCH-AS (see below). Combining *in situ* hybridization for MCH and immunohistochemistry for GFP, we verified that GFP-labeled perikarya correspond to MCH neurons (Figure 6).

**Figure 7:**
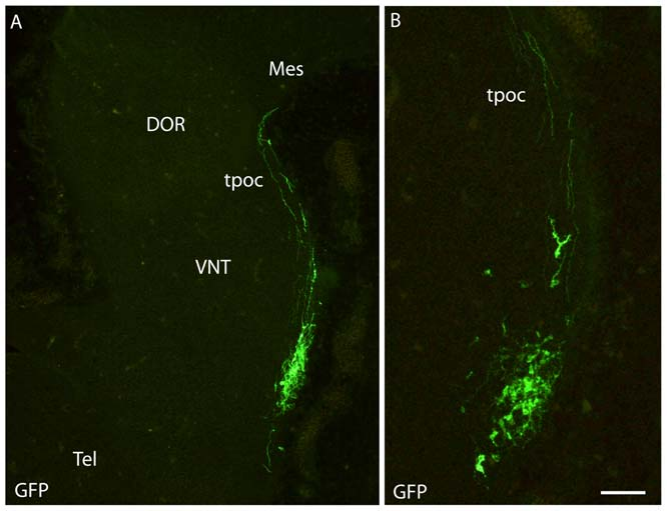
MCH-GFP perikarya and axons at E11. (A–B) On a sagittal section of E12 brain, MCH-GFP fibers follow the *tpoc*. Most MCH-GFP axons course dorsally/caudally within this tract in direction of the mesencephalon. B shows a higher magnification. Scale bar: A = 150 µm; B = 75 µm. DOR: thalamus dorsal; Tel: telencephalon; *tpoc*: *tractus postopticus*; Mes: mesencephalon; VNT: thalamus ventral.

**Figure 8:**
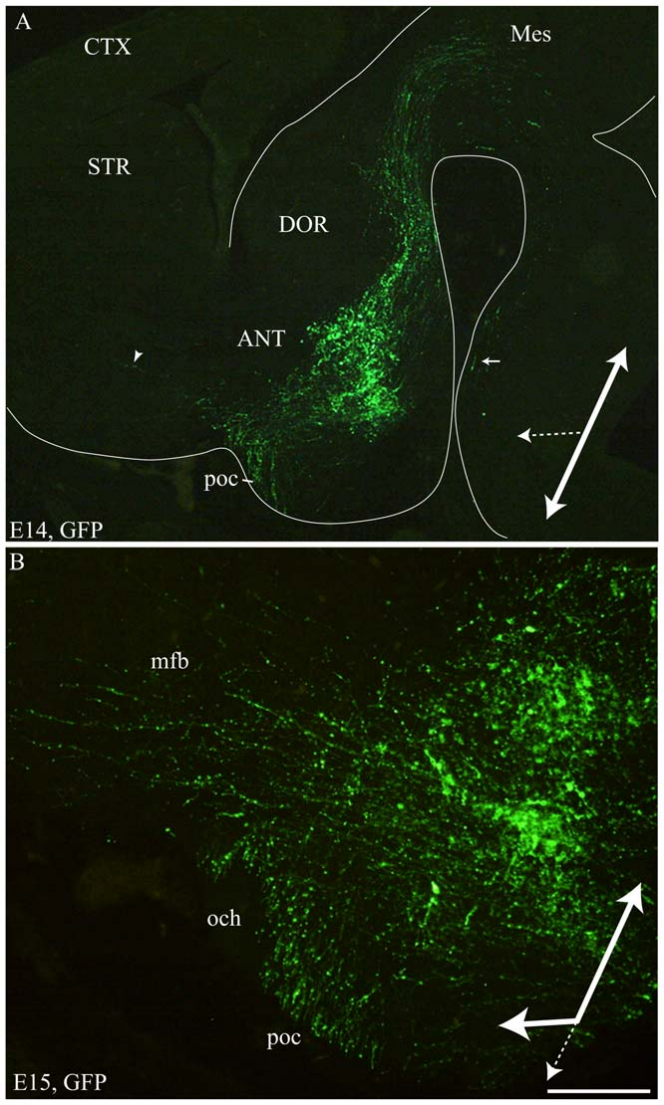
MCH-GFP perikarya and axons at E14–15. Photomicrographs of the GFP labeling on para-sagittal sections of E14 (A) and E15 (B) MCH-GFP embryos. Caudally directed GFP fibers form a thick tract reaching the mesencephalon. Some of these caudally directed axons are followed ventrally in the neural tube as far as the spinal cord (arrow in A). Another thick bundle of axons run toward the postoptic commisure (poc). Few axons (arrowhead in A) are observed in direction of basal telencephalon at E14 (A). (B) At E15, the number of GFP axons observed in the mfb toward the basal telencephalon dramatically increases. These observations suggest that at E14 most axons follow the tpoc (large white arrow), but some start to take a rostral route (large doted arrow). At E15, it seems that most axons are oriented caudally or rostrally, but less of them take a ventral route. Scale bar: A = 750 µm; B = 200 µm. ANT: anterior level, hypothalamus; CTX: cerebral cortex; DOR: dorsal thalamus; och: optic chiasm; STR: striatum.

**Figure 9:**
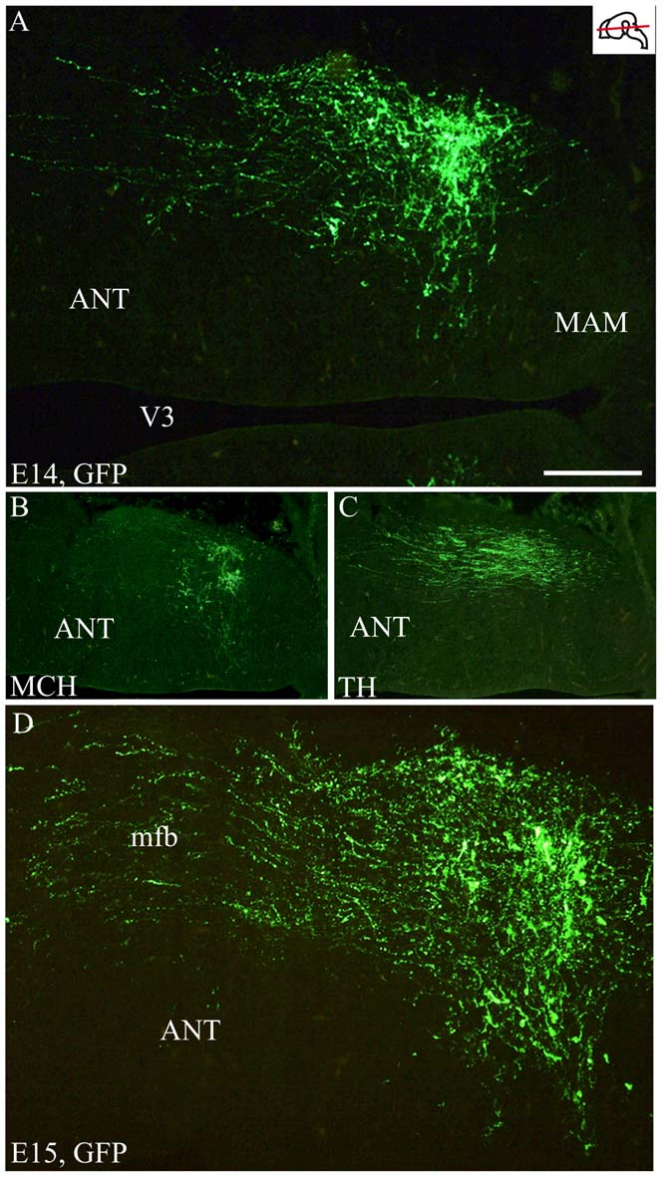
MCH-GFP perikarya and axons and TH axons at E14–15. Photomicrographs of the GFP labeling on horizontal sections of a E14 (A–C) and E15 (D) MCH-GFP embryos. (A–C) At E14, many MCH-GFP perikarya (A) are observed in the tuberal hypothalamus and some fibers take a rostral direction toward the telencephalon. On the ventrally adjacent section (B), MCH-AS labels perikarya, but axons are not detected. (C) On the section dorsally adjacent to A, a tyrosine hydroxylase (TH)-AS intensely labeled mesostriatal axons in the medial forebrain bundle (mfb). (D) At E15, rostrally directed axons are very abundant in the mfb. Scale bar: A, D = 200 µm; B, C = 500 µm. ANT: anterior level, hypothalamus; MAM: mammillary level hypothalamus; V3: third ventricle.

**Figure 10:**
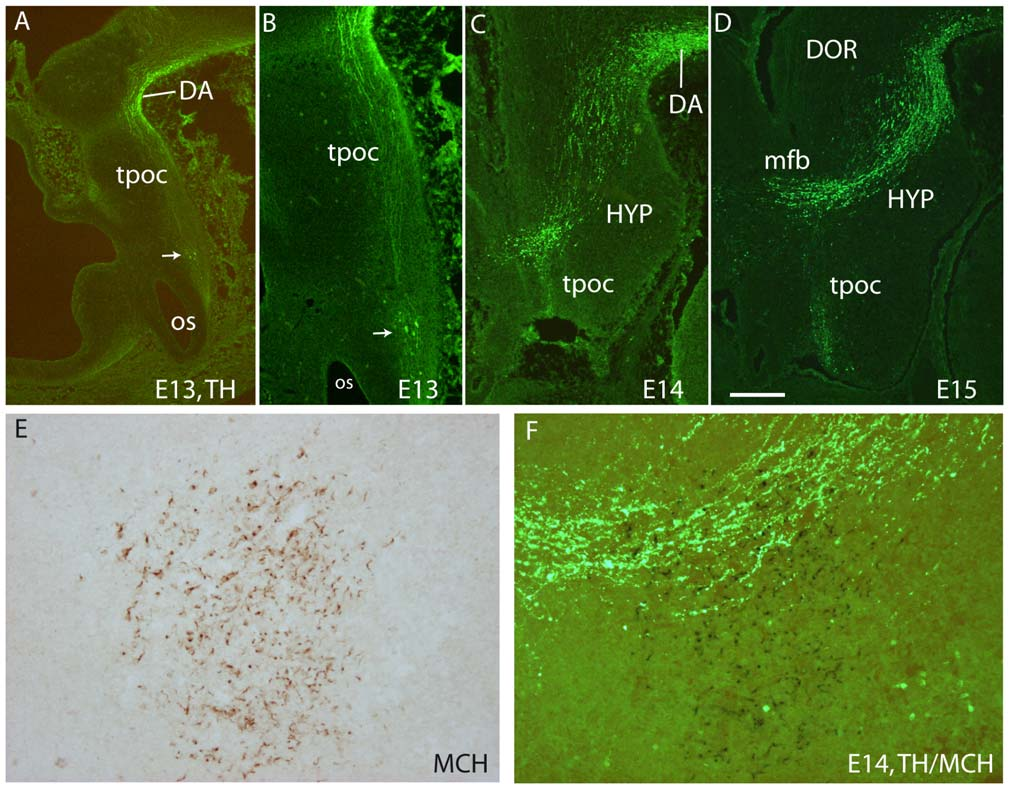
Spatiotemporal distribution of TH in E11 to E15 rat embryos. (A–D) Photomicrographs of para-sagittal sections through the diencephalon of E13 (A, B), E14 (C) and E15 (D) rat embryonic brains to illustrate the distribution of tyrosine hydroxylase (TH) immunofluorescence signal. At E13, cells (arrows in A and B) are labeled caudal to the optic stalk (os) and axons course in the direction of the midbrain through the *tpoc*. In the ventral midbrain, some cells are also detected (DA), but the mesotelencephalic tract is not yet visible. At E14 and E15, although cells and axons are still visible in the retrochiasmatic region, the mesotelencephalic axons form a prominent tract which first follows up the *tpoc*, and take a rostral route towards the telencephalon at the level of the caudal hypothalamus (HYP). (E, F) Photomicrograph of a para-sagittal E15 rat brain section labeled using immunoperoxidase to detect MCH (E) and immunofluorescence for TH (F). MCH cell bodies are within or close to the dopaminergic mesotelencephalic tract. See text for details. Scale Bar: A–D = 250 µm; E, F = 50 µm.

On sagittal or horizontal sections from E11 or E12 MCH-GFP mouse embryo, GFP antibody labeled axons originating from a small group of neurons very lateral in the mantle layer of the posterior hypothalamus (Figure 7). These axons were in the tpoc and most of them coursed caudally toward the mesencephalon that they had reached by E12 (Figure 7). A very few were also seen taking a ventral direction in the *tpoc* toward the postoptic commissure (poc). At later stages (E14 – Figure 8A), caudally directed axons formed a very thick bundle, arching in the mesencephalon and extending in the ventral caudal brainstem to reach the spinal cord. Another thick bundle of axons from the same cell group reached the postoptic commissure. However, axons were also seen passing above the optic chiasm in direction of the ventral telencephalon. At E15 (Figure 8B) or on horizontal sections (Figure 9D) these axons directed toward the telencephalon were abundant. Adjacent sections were labeled for MCH or tyrosine hydroxylase (TH) (Figure 9A–C). Interestingly, GFP perikarya were also observed very medially in the germinal layer, suggesting that MCH neurons migrate radially from the ventricular surface into the mantle layer (Figure 9A,D). To illustrate the course of the medial forebrain bundle on our material, some of its axons were labeled using antibodies raised against TH. The region containing MCH cell bodies exhibited a thick bundle of TH labeled axons, confirming our previous observations in rat that many MCH neurons lay within this tract already in the embryo [17]. This pathway corresponds to the mesotelencephalic tract, which initially follows up the *tpoc* from the ventral mesencephalon but arches rostrally at posterior hypothalamic levels to reach the telencephalon (Figure 10A–D). MCH neurons were labeled in the posterior hypothalamus adjacent to mesotelencephalic axons (Figure 10E, F).

Therefore, a clear correlation exists between the differentiation/direction of the *tpoc* and the differentiation/direction of first MCH neurons/projections. During later stages, another clear correlation exists between the differentiation of ascending (dopaminergic) components of the medial forebrain bundle (*mfb*) and differentiation of late generated MCH cells/rostrally directed MCH axons. At those stages, neither DiI nor GFP immunohistochemistry labeled other fiber tracts from the caudal hypothalamus, and we can assume that the *tpoc* and later the *mfb* form the first scaffold of the MCH-projection pattern.

### Mesencephalon and telencephalon influence the direction of MCH projections

**Figure 11:**
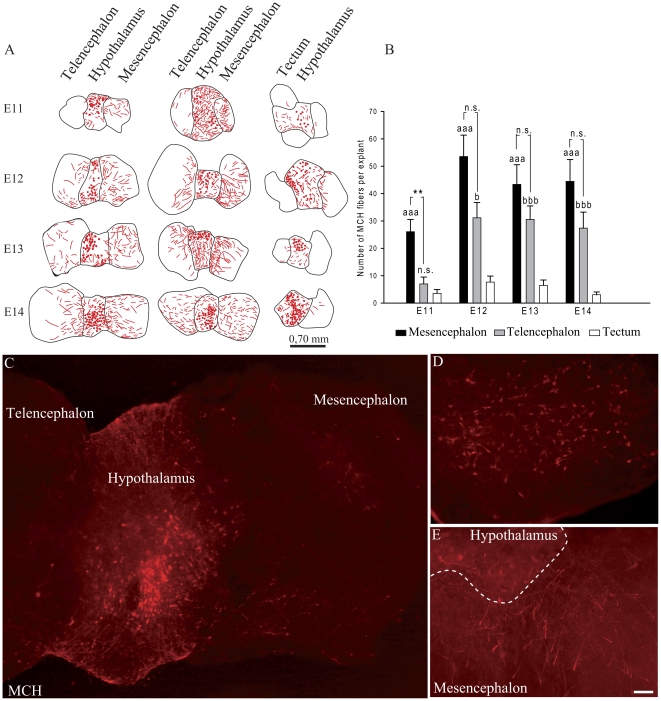
Mesencephalic or telencephalic influences on MCH axonal growth. (A) Series of line drawings of co-culture experiments. Red dots in hypothalamic explants represent MCH perikarya; red lines represent MCH fibers revealed by immunofluorescence. Note that the innervation of the telencephalic and/or mesencephalic explants is independent of the size of these explants and of the presence of MCH perikarya close to the corresponding edge in the hypothalamic explants. (B) Newborn mice caudal hypothalamic explants were co-cultured with E11 to E14 embryonic basal midbrain and basal telencephalic explants. Data were obtained from at least 3 independent experiments for each embryonic stage. 12 to 19 basal midbrain and basal telencephalic explants were analyzed per stages and 9 to 17 tectal explants that were used as controls. Data indicate mean ±SEM. aaa: p≤0,001 mesencephalon vs tectum; bbb: p≤0,001 - b: p≤0,05 telencephalon vs tectum; **: p≤0,01 mesencephalon vs telencephalon; n.s.: not significant, Kruskal Wallis's test. (C–E) Photomicrographs of slices after two days *in vitro* and MCH immunofluorescence. Low (C) and higher (D,E) magnifications to illustrate that MCH neurons survived very well, and that perikarya and axons are clearly labeled and can easily be numbered. Scale bar in E: C = 60 µm; D, E = 30 µm.

Immunohistochemical and DiI experiments suggested that the basal telencephalon attracts MCH axons at later stages than the brainstem. To verify this hypothesis, newborn mouse hypothalamic explants were co-cultured with E11 to E14 mesencephalic and basal telencephalic explants (Figure 11). After two days *in vitro*, MCH cell bodies and axons were revealed using immunocytochemistry (Figure 11C–E). MCH axons were abundantly observed in all mesencephalic explants. By contrast, E11 telencephalic explants and E11 to E14 tectal controls contained no or very few MCH axons. Furthermore, when such axons were present in these explants, they did not penetrate deep, but stayed at the periphery (Figure 11A). MCH axons were abundantly seen deeper into E12 to E14 telencephalic explants.

Labeled axons were counted in all embryonic explants and statistical analysis confirmed immunocytochemical observation (Figure 11B): MCH axons were significantly more abundant in E11 mesencephalic explants than in the age-matched telencephalic tissue. E11 telencephalic innervation was similar to the age-matched tectal controls. Conversely, telencephalic projections were significantly more abundant than in the age-matched controls when embryonic explants were taken at E12. This phenomenon was even more significant with E13/14 explants. By comparison, the innervation of E13/14 telencephalic explants matched the projections of corresponding mesencephalic explants.

### Netrin1 and Slit2 are involved in the guidance of MCH axons

**Figure 12:**
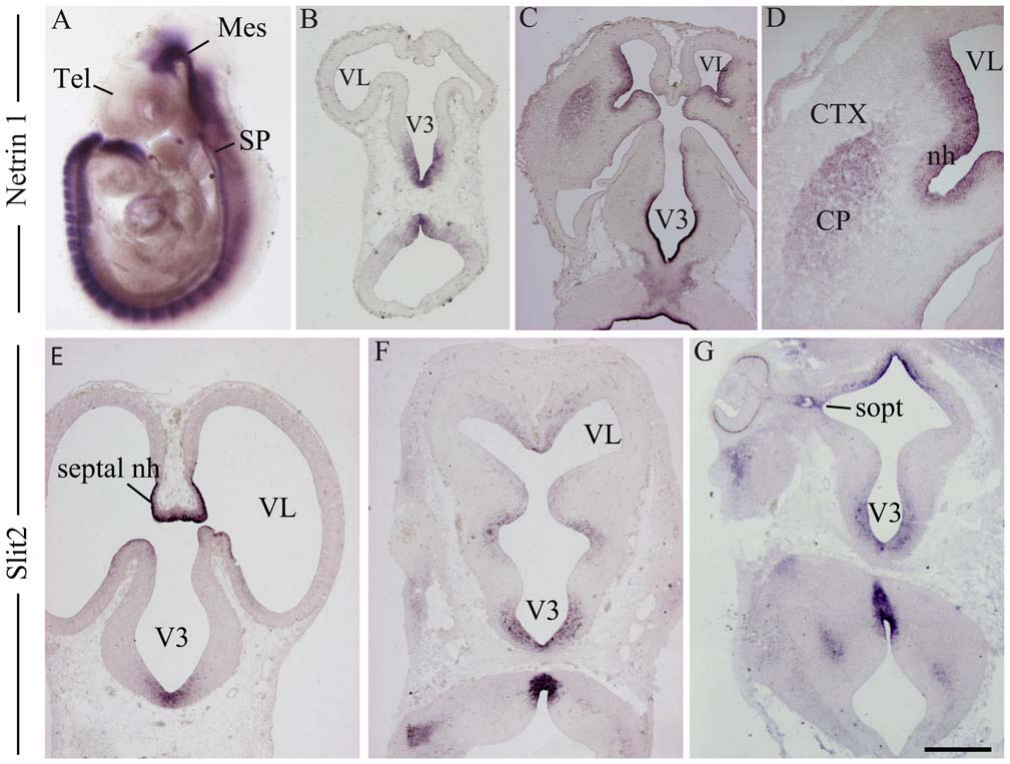
Expression of Netrin1 and Slit2. (A) Whole mount *in situ* hybridization to detect Netrin1 expression in an E9 mouse embryo. Note the absence of signal in the telencephalic vesicles. (B–D) Photomicrographs illustrating Netrin1 *in situ* signal on sections of E11 (B) and E14 (C, D) mouse embryonic brains. Netrin1 mRNA is detected in the telencephalon of E14 embryos, but in E11 embryos, telencephalic vesicles are not labeled. (E–G) Photomicrographs showing Slit2 *in situ* signal on horizontal sections of E13 (E, F) and E14 (G) rat embryos. Slit2 is expressed early in the septal neuroeptithelium (E13) and in the hypothalamic neuroepithelium (E, F). At E14, Slit2 is expressed in preoptic and posterior hypothalamic neuroepithelium (G). Scale bar: A = 1 mm; B, E–G = 400 µm; C = 600 µm; D = 250 µm. CP: caudoputamen; CTX: cerebral cortex; Mes: mesencephalon; nh: neuroepithelium; septal nh: septal neuroepithelium; sopt: optic sulcus; SP: spinal cord; Tel: telencephalon; V3: third ventricle; VL: lateral ventricle.

The attraction of posterior hypothalamic MCH axons by telencephalic or mesencephalic explants may depend of a timely expression of axonal guidance cues. Many factors are involved in the growth of the *tpoc* and/or in the attraction of axons by the telencephalon, but Netrin and Slit family members appear to play determinant roles. Netrin1 is the most studied member of the Netrin family. This molecule interacts with specific receptors to induce long- or short-range chemoattractive or chemorepulsive responses [31]. It is a key determinant of thalamo-cortical axonal patterning [32], [33] and is also involved in the guidance of the mesotelencephalic pathway [34], [35]. Slit2 is a chemorepulsive molecule that also plays a key role in the guidance of the dopaminergic mesotelencephalic tract [35], [36], [37], [38]. Both Netrin1 and Slit2 cooperate to guide the *tpoc* in the fish prosencephalon [39], [40]. Prior to E12 in the mouse embryo, Netrin1 mRNA is heavily expressed in the ventral midline of the neural tube caudal to the hypothalamus (Figure 12A–B). However, the *in situ* signal for Netrin1 was very weakly present or even barely detectable in the telencephalon. The *in situ* labeling of Netrin1 in both the germinal epithelium and mantle layer of the mouse ganglionic eminences increased dramatically after E12 [33], [41] (Figure 12C–D). Similar results were obtained in the rat embryo (data not illustrated). This observation suggested that higher levels of Netrin1 are expressed in the basal telencephalon only after neurogenesis has begun in this structure, which starts at E12/13 in the rat embryo [42]. By contrast, Slit2 *in situ* hybridization signal was constantly observed in the ventral midline of the neural tube (Figure 12E–G).

**Figure 13:**
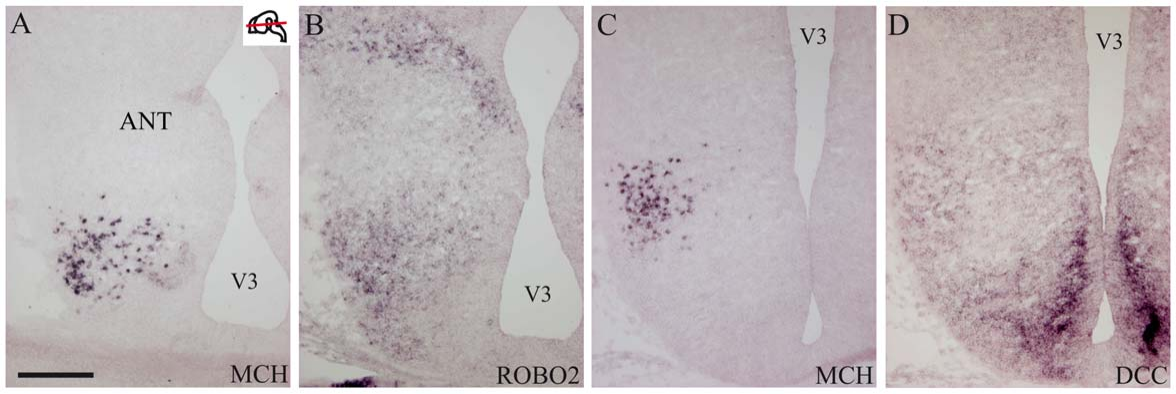
Expression of ROBO2, DCC and MCH. (A–D) Photomicrographs of adjacent horizontal sections passing through the hypothalamus of E15 rat embryos and labeled by *in situ* hybridization for MCH, DCC and Robo2. The MCH region contains intense DCC and Robo2 *in situ* signals. Scale bar = 300 µm.

**Figure 14:**
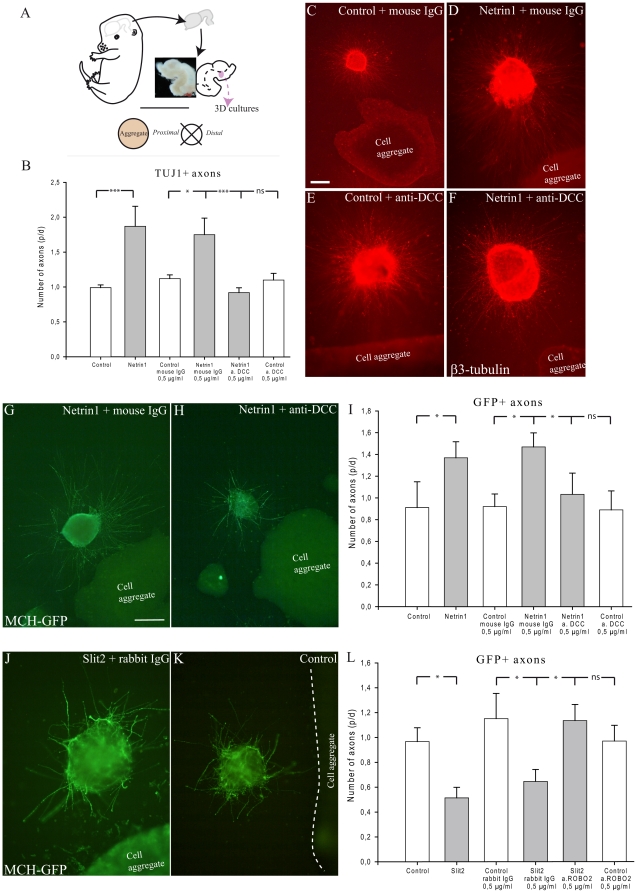
Netrin1 attracts and Slit2 repulses MCH axons. (A) Tridimensional culture experiments. E16 embryonic brains were dissected out. The ventral midline was incised to expose the ventricular surfaces. Posterior hypothalamic regions were isolated, cut into 200 µm thick pieces and deposited around cell aggregates. Quantification was performed as follow: after two days *in vitro*, each explant was divided into four quadrants. TUJ1 or GFP positive fibers were counted in the proximal and distal quadrants. The ratio between proximal and distal (p/d) fiber number was then calculated for each explant. (B) Statistical analysis: caudal hypothalamic axons were significantly attracted by Netrin1-transfected cells aggregates, but not by control cells. Addition of an anti-DCC antibody blocks the effect of Netrin1 on axons. (C–F): Netrin1 attracts axons of caudal lateral hypothalamus. Aggregates of control or Netrin1-expressing HEK-293 cells were confronted to caudal hypothalamic explants containing interneurons. Axons growth is visualized with the TUJ1 (anti-β3 tubulin) antibody. Netrin1 exerts a clear attractive effect compared to control cases. This effect is abolished by incubation with the anti-DCC antibody. (G, H) Photomicrographs of two explants labeled by the GFP-AS and cultured cell aggregates expressing Netrin1and incubated with control IgG or anti-DCC IgG. Netrin1 attracts GFP axons, but this effect is inhibited by anti-DCC IgG (I) Statistical analysis: MCH-GFP axons are attracted by Netrin1 but not by controls. (J, K) Slit2 repelled MCH-GFP axons. Aggregates of control or Slit2-expressing HEK-293 cells were confronted to caudal hypothalamic explants containing interneurons. Axons growth is visualized with the GFP antibody. Slit2 exerts a clear repulsive effect compared to control cases. This effect is abolished by incubation with the anti-Robo2 antibody. (L) Statistical analysis: MCH-GFP axons are repelled by Slit2 but not by controls. Data indicate mean ±SEM. ***: p≤0,001; *: p≤0.05; n.s.: not significant, Mann Whitney's test. Scale bar (C–F): C = 200 µm; D–F = 400 µm; Scale bar (G, H, J, K): G, H = 200 µm; J, K = 400 µm.

DCC and Robo2, respective Netrin1 and Slit2 receptors, are expressed in neurons of the posterior hypothalamus. Both DCC and Robo2 *in situ* signals were very intense in the MCH- containing region (Figure 13). DCC is involved in both chemoattractive and chemorepulsive responses to Netrin1 [31]. To determine influence of Netrin1 on MCH axon growth, we performed a guidance assay, in which MCH area explants from Swiss or MCH-GFP mice were co-cultured with aggregates prepared from 293-HEK cells transfected with Netrin1 or control plasmid in the plasma/thrombin gel for two days (Figure 14). After revelation with antibodies against β3-tubulin or GFP, quantitative analysis was performed by counting axons in the proximal and distal quadrants of the explants (Figure 14A). Ratios of numbers of axons in proximal versus distal area were then calculated (p/d ratio). We found that explants cultured with Netrin1 exhibited more neurite outgrowth than control explants. Neurites from explants cultured with control 293-HEK cells grew radially in all orientations (Figure 14C,K). Increased axonal growth toward to Netrin1 expressing cells was observed in explants cultured with Netrin1 aggregates (Figure 14D,G). Statistical analysis showed a significant increase of neurites distributed in the area facing Netrin1 cells compared to that in the control explants (Figure 14B, I). To evaluate whether the DCC receptor mediated such promoting effects, a functional blocking antibody against DCC (clone AF5) was applied at a concentration of 0.5 µg/ml. Addition of the species-matched immunoglobulin served as a control. Quantitative analysis revealed that presence of Netrin1 transfected cells aggregates significantly increased caudal hypothalamic or MCH-GFP neurite outgrowth when compared to that in explants cultured with control cells, with IgG and with the DCC antibody (Figure 14B, I). Netrin1 was therefore demonstrated to function as an attractant for axon growth through binding to the DCC receptor as in many regions in the developing brain [32], [43].

By contrast, MCH-GFP-containing explants cultured with Slit2 cell aggregates, most GFP positive axons were observed in the distal part of the explants, and only few axons were seen in the proximal aspect that faced the aggregates (Figure 14J–K). As above, quantification showed that more GFP-axons were distributed from the distal aspects of the explants cultured with Slit2 cell aggregates (Figure 14L), compared to control cell aggregates, indicating that Slit2 is a potent repellent for MCH axon growth. Slit2 action is mediated by Robo receptors, since addition of an Robo2 antibody (0.5 µg/ml) neutralizes Slit2 effects on MCH neurite extension in our study. Addition of the species-matched immunoglobulin served as a control (Figure 14J–L).

These findings clearly demonstrated that guidance molecules Netrin1 and Slit2 influence MCH axon outgrowth, indicating roles for these molecules in the formation of the MCH pathways.

## Discussion

### MCH neurons differentiate within a longitudinal neurogenic column

Differentiation of the mantle layer in the rat diencephalon begins at E11 in the retrochiasmatic region, soon after the anterior neuropore closure, and follows a dorsal progression toward the ventral thalamus [44]. This pattern of mantle layer emergence was fully described in past tritiated thymidine studies and reviewed by Keyser in the guinea pig [23], [24]. We also recognized this pattern in our rat material after E11 BrdU injections. MCH neurons differentiate within this area, and many of them contained BrdU after E11 injections. Then, first MCH cell bodies are clearly generated within this cell cord at this early stage, and are among the very first to be generated in the diencephalon, along with other unidentified cells.

An hypothalamic segment of this cell cord was recently named the Intrahypothalamica Diagonal on the basis of distribution of hundreds of genes in the mouse embryo [22], [26]. This diagonal lies along the Shh domain that extends longitudinally through the ventral neural tube. Nkx2.2 is expressed following a similar pattern [27]. MCH neurons differentiate in a specific part of the Nkx2.2 domain that expressed Nkx2.1, at the border of Pax6-expressing region. Expression of both Nkx2.1 and Nkx2.2 was observed in MCH neurons. Nkx family members are Shh controlled genes [45], [46]. This is coherent with the previous findings of Szabo et al. [29], corroborated in our study, that MCH differentiation in the caudal hypothalamus results from a Shh controlled genetic cascade.

The differentiation of the MCH region implicates other genes: Dlx1-2 is involved in the differentiation of prosencephalic GABAergic cells [47], [48], and their expression in the MCH area can be correlated to the GABAergic nature of MCH neurons in the adult animal [49], [50], [51]. The adult MCH region contains other GABAergic cell populations [52] and Dlx in the posterior hypothalamic anlage may also specify these neurons. The expression pattern of Lhx9 is restricted to the MCH expression area. However Lhx9 expression may correspond to Hcrt neurons [22] that are interspersed with MCH cell bodies in the adult lateral hypothalamus.

The expression of MCH neurons along the Nkx2.2 stripe of tissue was quite intriguing. Emerging evo-devo models suggest that conserved patterns of mediolateral regions extending from the head to the trunk are reflected by the expression of genes that set up the molecular anatomy of the neurectoderm [53], [54], [55]. Specific neuronal types emerge from these regions at specific antero-posterior molecular coordinates. Nkx2.2 is described as specifying the medial neurogenic column. In the spinal cord and caudal brainstem, neurons produced from this column (for example serotoninergic neurons) are formed by early delaminating neuroblasts and scaffold pioneer longitudinal axonal tracts, and particularly the medial longitudinal fasciculus (*mlf*). The serotoninergic phenotype differentiates under the control of Shh, Nkx2.2 and Nkx6.1 in the medial neurogenic column [55]. These neurons send descending and ascending axons pioneering the *mlf*. MCH neurons share with serotoninergic cells an early genesis within the Nkx2.2 expression domain. MCH neurons express Nkx2.1, and differentiation of the MCH phenotype is also Shh dependent. First MCH axons run in the *tpoc* which is the pioneer prosencephalic longitudinal tract. This tract joins the *mlf* in the brainstem and course parallel to the Shh/Nkx2.2 expression domain [27], [28]. First MCH neurons could then differentiate at prosencephalic/Nkx2.1 level of the medial neurogenic column.

### Differentiation of descending and ascending MCH projection patterns

Our sequential BrdU approach illustrated that cytodieresis occurs close to 10 h after BrdU is incorporated by mother cells during the S phase of their last cell cycle. Then, BrdU injections should be more related to the ‘conception time’ of a neuron than its true birth. First neurons that differentiate the MCH phenotype were observed adjacent to the pioneer caudally directed *tpoc* in rat and mouse embryos, at least two days after first MCH cells incorporated BrdU. First MCH axons enter and run through this tract in both species. MCH cortically projecting cells incorporated BrdU at E12/13 in the rat. Therefore, MCH cells ‘conceived’ at these stages differentiate as axons from the diencephalon and ventral mesencephalon are growing toward the telencephalon in which neurogenesis has started. Hence, MCH axonal growth is clearly correlated to the expansion of pioneer tracts, very early for axons that follow the *tpoc* in direction of the spinal cord, but later for axons that run in the mfb with mesotelencephalic projections in direction of the telencephalon.

### Netrin1 and Slit2 may guide MCH axons

Co-culture experiments showed that ventral midbrain explants attracted significantly MCH axons at early stages while the basal telencephalon had no effect. Telencephalic explants contained a number of MCH axons significantly different than the control (tectum) only when it has differentiated a mantle layer. Very importantly, tectal explants never significantly attracted MCH axons in our co-culture experiments; the E14 tectum also shows a differentiated mantle layer, but E14 tectal explants contained no more MCH axons than E11 tectal explants. This observation clearly suggested that attractive cues are expressed in the mesencephalon and E12 to E14 telencephalic explants, but not in the tectum or E11 telencephalon, attract MCH axons. Our next step was to identify some of these factors.

Shh, Netrins, Slits, Ephrins and Semaphorins govern the development of the *tpoc* and dopaminergic projections [35], [38], [56], [57], [58], [59], [60]. In particular, Netrins play an important role as chemoattractants for both diencephalospinal projections and dopaminergic telencephalic afferents [39], [40], [60], [61], [62], [63], [64]. In our study, MCH and DCC which is responsible for the chemoattractive response to Netrin1, were co-expressed in the same region. The very intense Netrin1 expression in the caudal ventral neural tube contrasts with the late telencephalic Netrin1 expression. Therefore, Netrin1 expression in the telencephalon is correlated to the onset of neurogenesis in this structure which occurs later than in the brainstem and spinal cord.

Tridimensional culture experiments showed that posterior hypothalamic axons and more specifically MCH axons, are attracted by Netrin1 and that this attraction is mediated by DCC. Extrahypothalamic source of Netrin1 could then be involved in the growth of MCH axons outside the hypothalamus [32], [33], [34]. However, it is not the sole guidance cue involved. For example, neuroendocrine projections are a main output of magnocellular neurons in the hypothalamic paraventricular nucleus. These neurons are produced in the Nkx2.2/Pax6 region just rostral to MCH area [65], [66]. Neuroendocrine projections are guided by locally produced Netrin1 in the ventral midline of the hypothalamus. However neuroendocrine neurons lack Robo expression which mediates the repulsive action of Slit proteins [67], [68]. Therefore, Slit proteins, and in particular Slit2, are other essential actors to wire the hypothalamus. In Nkx2.1 mutants, Slit2 expression is down regulated in the hypothalamic neuroepithelium, and dopaminergic axons are not confined to the mesotelencephalic tract but innervate ventral hypothalamic structures [36]. This and other Slit proteins also channel axons of the *tpoc* that exit the hypothalamus [63]. MCH neurons are found in a Robo2 rich region. Furthermore our tridimensional culture experiments showed that MCH axons are repulsed by Slit2 and that this action is Robo2 dependent. MCH axons may be forced to follow the *tpoc* and/or the mfb, depending of the embryonic stage. Interestingly, a recent report also indicated that the MCH peptide itself may also affect axonal growth [69].

### The MCH system development enlightens a switch in the forebrain basic plan

**Figure 15:**
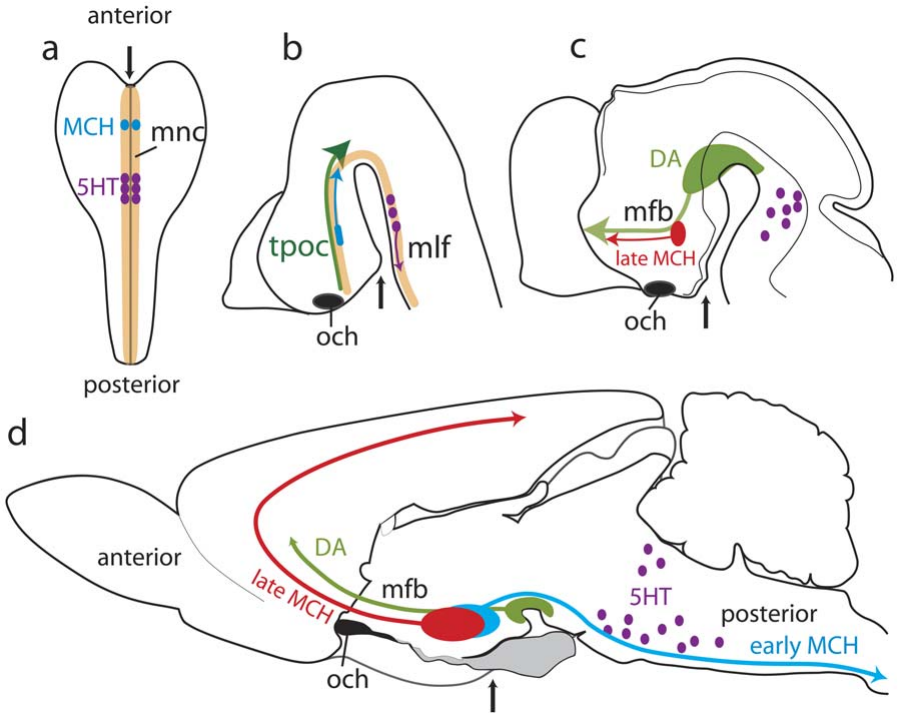
MCH development enlightens a switch in the prosencephalic basic plan. The medial neurogenic column (mnc) characterized in the embryo by the longitudinal expression of Nkx2.2, extends from the anterior to posterior pole of the neural plate (a). Specific neuronal phenotypes will be produced at specific stages of the mnc; serotoninergic neurons (dark purple dots) in the presumptive hindbrain, and MCH phenotype (blue dots) in the presumptive prosencephalon. After neural tube closure (b), first MCH neuroblasts (blue) are effectively produced. They send their axons through the *tpoc* that run parallel to the Nkx2.2 expression domain (dark green arrow show the direction of the tpoc). At the same stage, serotoninergic neurons (purple) differentiate and send their axons in the medial longitudinal fasciculus (*mlf*). Later (c), as neurogenesis begins in the telencephalon, axons from late produced MCH neurons (red) extend toward this structure. At the same stage, mesencephalic dopaminergic neurons (DA - light green area and arrow) differentiate, and their projections gather in a dense bundle in the mfb with MCH axons. In the adult animal (d), the mfb is the main longitudinal tract of the ventral diencephalon, connecting the rostral brainstem with the telencephalon, and defining the anteroposterior axis of the adult forebrain. The thin black arrows point the presumptive (a, b) or the anatomical position (c, d) of the pituitary which is at the rostral pole of the neural plate, but ventral surface of the adult brain.

Collectively, our results show that MCH neurons differentiate within a longitudinal region named ‘cell cord’ or ‘Intrahypothalamica Diagonal’. This region may as well be seen as a prosencephalic component of the early medial neurogenic column which extends through the ventral neural tube (Figure 15). MCH neurons share many characteristics with other neurons from this column, including an early genesis and axons coursing through a pioneer longitudinal tract. Neurogenic columns are conserved through the phylogenetic scale of vertebrates and even in bilaterian invertebrates. Conformingly, MCH neurons are observed in the caudal/dorsal hypothalamus of all vertebrates investigated so far, and are also described in the optic lobes of the *locusta* cephalic ganglia [70]. In the adult mammalian forebrain, vestiges of this early organization can be found in the path of the supraoptic commissures, optic chiasm and tract which follow the course of the *tpoc* toward the midbrain.

However, the differentiation of ascending MCH projections is correlated to an intense neurogenic activity in the dorsal telencephalon, as well as to the differentiation of ascending projections from the ventral midbrain. Thalamo-cortical bidirectional connections take place during the same period. Then, the growth of ascending MCH projections, along with others, is the result of a change in prosencephalic axial organization, with MCH and dopaminergic axons rostrally directed toward the telencephalon. These pathways constitute the medial forebrain bundle and correspond to the definitive longitudinal rostro-caudal axis of the adult forebrain (Figure 15).

To conclude, first generated MCH neurons may be influenced by an early organization scheme, that also pattern retinian input in the brain, but late produced cells follow an another scheme that pattern basal telencephalic (ventral striatal, and also maybe olfactory) networks. It is not clear yet if this dual developmental principle explains the various functions as sleep/wake cycle and food intake in which the MCH neuron population as whole is involved.

## Materials and Methods

### Animals and tissue treatment

All animal use and care protocols were in accordance with institutional guidelines (all protocols were approved and investigators authorized). All experiments were approved by the institutional ethic committee (protocol number: 10003). Long Evans rats were obtained from Charles River Laboratories, L'Arbresle, France, and Swiss mices from Janvier, Le Genest-Saint-Isle, France. Shh mutant mice maintained on a C57BL6/J background were obtained from Dr P. Kim (Toronto, Canada). Shh mutant mice have a deletion in the exon 2 region which has been replaced by a fragment containing a Pgk-neo cassette [71]. The genotyping of mutant mice Shh was done by PCR (Primers 5′ gAAgAGATCAAggCAAgCTCTggC 3′ and 5′ ggACACCATTCTATgCAggg 3′ and 5′ ATgCTggCTCgCCTggCTgTggAA 3′). MCH-GFP mice were obtained from Prof. J. M. Friedman (The Rockfeller University, N.Y.), these mice were maintained on a C57BL6 background [72], [73], [74].

The time of conception for rats was documented by sperm-positive vaginal smear examined on the morning following the mating night, or by a vaginal plug for mice (embryonic day 0, E0). The day of birth was considered postnatal day 0 (P0). Timed-pregnant rats and mice were anesthetized with an intraperitoneal injection (IP) of 7% chloral hydrate (1 ml per 200 g body weight, Prolabo). Mice pups were sacrified by decapitation. For *in situ* hybridization and immunohistochemistry, embryos were fixed with 1 or 4% paraformaldehyde in 0.1 M phosphate buffer (PB), pH 7.4 at 4°C. They were cryoprotected in 15% sucrose and frozen or progressively dehydrated in ethylic alcohol, embedded in paraffin before to be sectioned (10 or 16 µm thick).

Nomenclature and cytoarchitectonic divisions are mostly based on ‘Developmental Brain Maps: Structure of the Embryonic Rat Brain’ [44]. Sections were analyzed on an Olympus fluorescence microscope BX51 or an Olympus confocal microscope Fluoview FV1000 BX configuration. Images were obtained through a DP50 or DP75 numeric camera (Olympus, France), using the analySIS or Fluoview FV1000 softwares (Olympus, France).

### Bromodeoxyuridine injections

Pregnant rats (from gestational day 11 and 12.5) were given an IP injection of 5-bromo-2′-deoxyuridine (BrdU, Sigma, France; 160 mg/kg body weight dissolved in 0.07 M NaOH and warmed to 65°C) [75]. Embryos of the E11 BrdU-injected animals were removed 2 hours, 6 h, 10 h, 1 day (E12), 2 days (E13) or 3 days (E14) after injection. Embryos of the E12.5 BrdU injected animals were removed 1.5 (E14) days after injection. Two pregnant rats received four BrdU injections between E11–E11.5 and E12.5–E13 and both were sacrified at E14. For the detection of BrdU, an acid hydrolysis was first performed and sections were counterstained by DAPI.

### Immunohistochemistry

Immunohistochemical procedures were performed as already extensively described [15], [16], [17]. A restoration of antigenic sites was necessary for detection of MCH. Sections were incubated at 80°C with DAKO Target Retrieval Solution (DAKO) diluted at the 1∶10. Used primary antibodies, their suppliers, and dilutions can be found in Table 2 [76], [77]. The specificity of the salmon MCH-antiserum (AS) was verified by liquid phase inhibition, dot blot, and immuno-affinity [78], [79] as well as by immunohistochemistry/*in situ* hybridization double labeling [80], [81]. The labeling was then revealed through the standard peroxidase anti-peroxidase procedure (Dako, France) or indirect immunofluorescence procedures.

**Table 2:** Used Antibodies.

Antibody	Supplier	Dilution
Rabbit anti-sMCH	[79]	1∶200
Rabbit anti-TH	Jacques Boy	1∶1000
Rabbit anti-Dlx1-2	A kind gift from Dr G. Boekhoff-Falk, University of Wisconsin-Madison, [76]	1∶400
Rabbit anti-Pax6	Covance	1∶300
Rabbit anti-TTF1	Santa Cruz Biotechnology Inc	1∶200
Mouse anti-Nkx2.2	Developmental Studies Hybridoma Bank	1∶10
Mouse anti-neurofilament	Boehringer Mannheim	1∶500
Mouse anti-βIII tubulin	Covance (TuJ1)	1∶5000
Mouse anti-DCC	Calbiochem (AF5)	0.5 µg/ml
Rabbit anti-Robo2	Dr F. Murakami [77]	0.5 µg/ml
Rabbit anti-GFP	Millipore	1∶500 or 1∶3000
Mouse anti-BrdU	Becton Dickinson	1∶100

### *In situ* hybridization

Dlx1-2 RNA probe was given by Dr M. Wassef [82], Shh RNA probe was given by Dr C.C. Hui [83], Netrin1 RNA probe was given by Dr H. Takahashi [84], Slit2 and Robo2 RNA probe were given by Dr A. Chédotal [85] and MCH RNA probe was obtained in our laboratory [17]. The rat DCC cDNA was obtained by reverse transcription/polymerase chain reaction from total RNA of adult rat brain following a protocol described by Brischoux et al. [17]. The resulting fragment with 1053 pb corresponded to nucleotides 2919–3971 of the DCC mRNA.

The antisense and control sense probes were produced by using the RNA transcription kit (Roche) and were DIG-UTP-labeled. The recombinant plasmids containing the DCC cDNA insert were linearized with NcoI (Invitrogen) and transcribed by SP6 RNA polymerase. Briefly, the transcription reaction reagents were added as follows: 2 µL 10× transcription buffer, 2 µl NTP (10 mM containing 3.5 mM of DIG-11-UTP), 1 µg of the linearized template DNA, 20 U RNasin (ribonuclease inhibitor), 20 U of SP6 polymerase. The reaction ran at 37°C for 2 h in a waterbath and was stopped by adding 2 µl EDTA (0.2 M, pH 8).

Frozen sections were post-fixed in 4% paraformaldehyde in 0.1 M PB and rinsed with 0.1 M PB then 5× SSC then incubated for 2 h in prehybridization buffer at 56°C. After rinsing in 0.2× SSC, the sections were incubated overnight at various temperatures depending of the riboprobe (56°C for MCH, DCC; 60°C for Netrin1, Dlx1-2, Shh; 72°C for Slit2, Robo2), in humid chambers, with 50 µl hybridization buffer containing 5% Denhardt's and 50 or 100 ng labeled RNA probes. After rinsing with 5× SSC, sections were incubated successively in 0,2× SSC at 56°C (1 h30) and 0.2× at room temperature (5 min). They were incubated in anti-digoxigenin Fab fragments conjugated to alkaline phosphatase (1∶1300, overnight) and revealed with enzyme substrate NBT-BCIP added 10% PVA (Sigma) and 5 mM MgCl_2_ (overnight, at room temperature, RT).

Control hybridization, including hybridization with sense digoxigenin-labeled riboprobes was realized.

### Whole mount *in situ* hybridization

E9 embryos were fixed in 4% PFA at 4°C overnight. Then, embryos were dehydrated into methanol using a graded methanol/PBTween series (Phosphate Buffer Tween 1%). After rehydratation, embryos were bleached with 6% hydrogen peroxide in PBTween for 1 hour then treated with proteinase K (10 µg/ml). Embryos were washed in 2 mg/ml glycine in PBTween, then in PBTween. After postfixation with 4% PFA/0.2% glutaraldehyde, embryos were incubated in hybridization solution (50% formamide, SSC 5× pH 4.5, 1% SDS, 50 µg/ml salmon tRNA and 50 µg/ml heparin). Embryos were incubated overnight with 200 ng of RNA probe at 60°C. After rinsing with 50% formamide, 5× SCC pH 4.5, 1% SDS then with 50% formamide, 2× SCC pH 4.5, embryos were incubated in anti-digoxigenin Fab fragments conjugated to alkaline phosphatase (1∶2000) overnight at 4°C and revealed with NBT-BCIP.

### DiI tracing

Brains of E11 and E14 mouse embryos were dissected and fixed by immersion in 4% PFA in PB, pH 7.4. A DiI crystal was applied in the posterior hypothalamus or ventral midbrain. Brains were stored overnight in fixative at 37°C, rinsed in PBS. E11 whole brains and E14 brains cryostat-sectioned sagittaly or horizontally at 16 µm were examined by epifluorescence (Olympus SZX9 microscope).

### Organotypic brain slice culture

Brains from Swiss postnatal mice (p3 at p5) were dissected out and cut in 250 µm thick frontal section using a tissue chopper. The posterior hypothalamus was cut in blocs (±1 mm^2^). These explants were maintained in 2 ml of co-culture medium consisting of 50% Eagle's basal medium, 25% Hank's balanced salt solution and 25% horse serum supplemented with glutamine (2 mM), glucose (6.5 g/l) and antibiotics. Basal telencephalon, ventral midbrain and tectum of E11 to E14 mice embryos were used. Embryonic brains were embedded in 3% low melting-point agar and sectioned horizontally at 300 µm using a vibratome. Sections containing pallidal region, ventral midbrain or tectum were collected and region of interest dissected. One telencephalic explant and one age matched ventral midbrain explant were deposited adjacent but on opposite side of the hypothalamic tissue on membrane (Millicell-CM, 0.4 µm, Millipore). Membranes and explants were then placed in 35 mm dishes and cultured in the same culture medium for 48 hours at 37°C in 5% CO2. The relative orientation of hypothalamic and embryonic explants was random. MCH fibers and neurons were revealed by immunohistochemistry. Briefly, after fixation (PFA 4%, 30 minutes), explants were rinsed in 0.03% Triton X – 100 in 0.1 M PB (PBT, three times for 10 minutes each time; RT) then they were incubated with the primary antibody (anti –sMCH, 1∶1000). After washing in PBT (three times, 10 minutes each; RT), explants were incubated with the secondary antibody for 2 hours under gentle agitation (Cy3 donkey anti-rabbit, 1∶800), washed in PBT and observed. The number of visible MCH fibers was evaluated for each explant (telencephalon, mesencephalon, tectum). Data were obtained from at least 3 independent experiments for each embryonic stage. 12 to 19 basal midbrain and basal telencephalic explants were analyzed and 9 to 17 tectal explants were used as controls per stages. Data are given as mean ± SEM, and the statistical significance was assessed by the Kruskal Wallis's test.

### Tri-dimensional culture

#### Aggregates of Netrin1 expressing cells

Netrin1 expressing cells were provided by Dr E. Soriano [86]. Slit2-GFP expressing cells were prepared by selecting stable expressing clones in the presence of Geneticin following transfecting HEK-293 cells [87] with a CS2-based vector containing human Slit2 open reading frame fused at its carboxyl terminus with enhanced GFP together with a IRES-neo cassette. Individual clones were selected and tested for stable expression by Western blotting even when Geneticin was not added. Aggregates were generated as previously described. Briefly, HEK-293 cells stably transfected with a construct encoding *Netrin1-c-myc* or Slit2-EPE or with the vector alone [43], [86] were cultured in DMEM containing 10% fetal bovine serum, 2 mM glutamine, 200 µg/ml Hygromycin (Invivogen), 250 µg/ml Geneticin (Invivogen) and antibiotics for Netrin1 cells and the same without Geneticin for Slit2 cells. The HEK-293 cell lines transfected with control vector plasmid was used as control. The cells were cultured in 100×20 mm culture dishes with the growth media, then harvested, centrifuged and re-suspended in 200 µl medium. Cell aggregates were prepared by pipetting 20 µl cell containing medium on the lid of 100×20 mm culture dishes and cultured with DMEM during at least 12 h.

#### Experiments and Data analysis

Explants of the posterior hypothalamic region (MCH area) were prepared from E16 Swiss-CD1 embryos or from E15 MCH-GFP embryos. This area was dissected out and cut into small squares of approximatively 200 µm/side in Gey's balanced salt solution supplemented with glucose (6.5 g/l). Explants were maintained in 2 ml of culture medium consisting of 50% Neurobasal medium, 2% B27, glucose (6.5 g/l) and antibiotics. The same procedures were used to prepare explants for all assays. Aggregates and hypothalamic explants were placed in 20 µl chicken plasma (Sigma) on a glass coverslip in 35 mm dishes. During coagulation of the clot with 20 µl thrombin (Sigma), explants were arranged around aggregates at 100–500 µm distances. After 45 minutes, 2 ml of culture medium were added and these dishes were transferred to the incubator (37°C, 5% CO2). After 48 hours, outgrowth was sufficient. Cultures were fixed in 4% PFA, 3% sucrose and immunostained with an antibody against neuron-specific class III β-tubulin (clone TUJ1, 1∶5000; Table 2) or antibody against GFP (1∶3000; Table 2). Blocking experiments were carried out by co-culturing Swiss CD1 or MCH-GFP hypothalamic explants and Netrin1-producing cells in the presence of 0.5 µg/ml of anti-DCC mAb (clone AF5, Table 2) or control mouse IgG. The same experiments were realized by culturing these explants and Slit2-producing cells in the presence of 0.5 µg/ml of anti-Robo2 (Table 2, generously provided by Dr F. Murakami, Japan) or control rabbit IgG. Immunostained explants cultures were examined under OLYMPUS microscope (Olympus BX51). In addition, a few explants cultures were immunostained for Nkx2.1 to confirm the identity of the posterior hypothalamus explants. To quantify effects of Netrin1 and Slit2, we determined number of axons by counting individual fibers. Each explant was divided into four quadrants. For E16 Swiss CD1 hypothalamic explants, TUJ1 positive fibers that crossed a line placed at a distance of 60 µm from the limit of the explants were counted in proximal (in front of cell aggregate) and distal quadrants. For E15 MCH-GFP hypothalamic explants, GFP positive fibers were counted in the same protocol. A ratio between proximal and distal (p/d) fiber number was calculated. Data (±SEM) were obtained from 5 at 7 independent experiments and between 11 and 26 Swiss CD1 explants and between 3 and 5 independent experiments and between 6 and 16 MCH-GFP explants and were analyzed for each condition. Statistical analysis was done using the Mann Whitney's test.

### Gene expression analysis

Three experiments were implemented to verify that pMCH gene expression is Shh dependent: pMCH mRNA was quantified from 1 Shh +/+ and 1 Shh −/− head of E13 embryos. The Shh pathway inhibitor cyclopamine (6 mg/Kg) was injected in E11 pregnant mice. pMCH mRNA was quantified in the head of 4 E13 embryos and compared to that of 4 control (DMSO injected E11 pregnant mice). Half whole brains of E11 mice embryos were cultured in presence of cyclopamine (20 µM) (7 brains) or DMSO (3 brains). The ventricular side faced the filter. pMCH mRNA was then quantified after 2 days *in vitro*. Total RNA was extracted using TRI REAGENT with DNAse (Fermentas) treatment following the manufacturer's instructions. Then, cDNA was synthesized from 1 µg total RNA using oligo(dT), dNTP and MMLV Reverse transcriptase (Fermentas). Real-time PCR was performed with gene specific primers for pMCH [88]. 1 µl aliquot of 1∶ 10 diluted cDNA was subjected to real-time RT-PCR using SyBR Green One step RT-PCR reagents (Applied Biosystems). All reactions were run in triplicate (Applied Biosystems), and results were normalized by β-actin expression. The forwards primer was 5′ TggTgggAATgggTCAgAAg 3′ and the reverse primer was 5′ TCCATgTCgTCCCAgTTggT 3′. Data are given as mean ± SD and statistical analysis was done using Kruskal Wallis's test.

## References

[1] Bittencourt JC, Presse F, Arias C, Peto C, Vaughan J (1992). The melanin-concentrating hormone system of the rat brain: an immuno- and hybridization histochemical characterization.. J Comp Neurol.

[2] Hanriot L, Camargo N, Courau AC, Leger L, Luppi PH (2007). Characterization of the melanin-concentrating hormone neurons activated during paradoxical sleep hypersomnia in rats.. J Comp Neurol.

[3] Hassani OK, Lee MG, Jones BE (2009). Melanin-concentrating hormone neurons discharge in a reciprocal manner to orexin neurons across the sleep-wake cycle.. Proc Natl Acad Sci U S A.

[4] Steinbusch HW (1991). Distribution of histaminergic neurons and fibers in rat brain. Comparison with noradrenergic and serotonergic innervation of the vestibular system.. Acta Otolaryngol Suppl.

[5] Steinbusch HW, Sauren Y, Groenewegen H, Watanabe T, Mulder AH (1986). Histaminergic projections from the premammillary and posterior hypothalamic region to the caudate-putamen complex in the rat.. Brain Res.

[6] Kayama Y, Koyama Y (1998). Brainstem neural mechanisms of sleep and wakefulness.. Eur Urol.

[7] Peyron C, Tighe DK, van den Pol AN, de Lecea L, Heller HC (1998). Neurons containing hypocretin (orexin) project to multiple neuronal systems.. J Neurosci.

[8] Qu D, Ludwig DS, Gammeltoft S, Piper M, Pelleymounter MA (1996). A role for melanin-concentrating hormone in the central regulation of feeding behaviour.. Nature.

[9] Griffond B, Risold PY (2009). MCH and feeding behavior-interaction with peptidic network.. Peptides.

[10] Griffond B, Baker BI (2002). Cell and molecular cell biology of melanin-concentrating hormone.. Int Rev Cytol.

[11] Conductier G, Nahon JL, Guyon A (2011). Dopamine depresses melanin concentrating hormone neuronal activity through multiple effects on alpha2-noradrenergic, D1 and D2-like dopaminergic receptors.. Neuroscience.

[12] Chung S, Hopf FW, Nagasaki H, Li CY, Belluzzi JD (2009). The melanin-concentrating hormone system modulates cocaine reward.. Proc Natl Acad Sci U S A.

[13] Shirayama Y, Chaki S (2006). Neurochemistry of the nucleus accumbens and its relevance to depression and antidepressant action in rodents.. Curr Neuropharmacol.

[14] Presse F, Nahon JL (1993). Differential regulation of melanin-concentrating hormone gene expression in distinct hypothalamic areas under osmotic stimulation in rat.. Neuroscience.

[15] Brischoux F, Cvetkovic V, Griffond B, Fellmann D, Risold PY (2002). Time of genesis determines projection and neurokinin-3 expression patterns of diencephalic neurons containing melanin-concentrating hormone.. Eur J Neurosci.

[16] Cvetkovic V, Brischoux F, Jacquemard C, Fellmann D, Griffond B (2004). Characterization of subpopulations of neurons producing melanin-concentrating hormone in the rat ventral diencephalon.. J Neurochem.

[17] Brischoux F, Fellmann D, Risold PY (2001). Ontogenetic development of the diencephalic MCH neurons: a hypothalamic ‘MCH area’ hypothesis.. Eur J Neurosci.

[18] Risold PY, Croizier S, Legagneux K, Brischoux F, Fellmann D (2009). The development of the MCH system.. Peptides.

[19] Croizier S, Franchi-Bernard G, Colard C, Poncet F, La Roche A (2010). A comparative analysis shows morphofunctional differences between the rat and mouse melanin-concentrating hormone systems.. PLoS One.

[20] Skofitsch G, Jacobowitz DM, Zamir N (1985). Immunohistochemical localization of melanin concentrating hormone-like peptide in the rat brain.. Brain Res Bull.

[21] Zamir N, Skofitsch G, Jacobowitz DM (1986). Distribution of immunoreactive melanin-concentrating hormone in the central nervous system of the rat.. Brain Res.

[22] Shimogori T, Lee DA, Miranda-Angulo A, Yang Y, Wang H (2010). A genomic atlas of mouse hypothalamic development.. Nat Neurosci.

[23] Keyser A (1972). The development of the diencephalon of the Chinese hamster. An investigation of the validity of the criteria of subdivision of the brain.. Acta Anat Suppl (Basel).

[24] Keyser A, Morgane PJ, Pankseep J (1979). Development of the hypothalamus in mammals, An investigation into its morphological position during ontogenesis.. Handbook of the hypothalamus Anatomy of the hypothalamus.

[25] Taupin P (2007). BrdU immunohistochemistry for studying adult neurogenesis: paradigms, pitfalls, limitations, and validation.. Brain Res Rev.

[26] Diez-Roux G, Banfi S, Sultan M, Geffers L, Anand S (2011). A high-resolution anatomical atlas of the transcriptome in the mouse embryo.. PLoS Biol.

[27] Shimamura K, Hartigan DJ, Martinez S, Puelles L, Rubenstein JL (1995). Longitudinal organization of the anterior neural plate and neural tube.. Development.

[28] Vokes SA, Ji H, McCuine S, Tenzen T, Giles S (2007). Genomic characterization of Gli-activator targets in sonic hedgehog-mediated neural patterning.. Development.

[29] Szabo NE, Zhao T, Cankaya M, Theil T, Zhou X (2009). Role of neuroepithelial Sonic hedgehog in hypothalamic patterning.. J Neurosci.

[30] Mastick GS, Easter SS (1996). Initial organization of neurons and tracts in the embryonic mouse fore- and midbrain.. Dev Biol.

[31] Barallobre MJ, Pascual M, Del Rio JA, Soriano E (2005). The Netrin family of guidance factors: emphasis on Netrin-1 signalling.. Brain Res Brain Res Rev.

[32] Braisted JE, Catalano SM, Stimac R, Kennedy TE, Tessier-Lavigne M (2000). Netrin-1 promotes thalamic axon growth and is required for proper development of the thalamocortical projection.. J Neurosci.

[33] Powell AW, Sassa T, Wu Y, Tessier-Lavigne M, Polleux F (2008). Topography of thalamic projections requires attractive and repulsive functions of Netrin-1 in the ventral telencephalon.. PLoS Biol.

[34] Vitalis T, Cases O, Engelkamp D, Verney C, Price DJ (2000). Defect of tyrosine hydroxylase-immunoreactive neurons in the brains of mice lacking the transcription factor Pax6.. J Neurosci.

[35] Lin L, Rao Y, Isacson O (2005). Netrin-1 and slit-2 regulate and direct neurite growth of ventral midbrain dopaminergic neurons.. Mol Cell Neurosci.

[36] Marin O, Baker J, Puelles L, Rubenstein JL (2002). Patterning of the basal telencephalon and hypothalamus is essential for guidance of cortical projections.. Development.

[37] Kawano H, Horie M, Honma S, Kawamura K, Takeuchi K (2003). Aberrant trajectory of ascending dopaminergic pathway in mice lacking Nkx2.1.. Exp Neurol.

[38] Dugan JP, Stratton A, Riley HP, Farmer WT, Mastick GS (2011). Midbrain dopaminergic axons are guided longitudinally through the diencephalon by Slit/Robo signals.. Mol Cell Neurosci.

[39] Devine CA, Key B (2008). Robo-Slit interactions regulate longitudinal axon pathfinding in the embryonic vertebrate brain.. Dev Biol.

[40] Kastenhuber E, Kern U, Bonkowsky JL, Chien CB, Driever W (2009). Netrin-DCC, Robo-Slit, and heparan sulfate proteoglycans coordinate lateral positioning of longitudinal dopaminergic diencephalospinal axons.. J Neurosci.

[41] Metin C, Deleglise D, Serafini T, Kennedy TE, Tessier-Lavigne M (1997). A role for netrin-1 in the guidance of cortical efferents.. Development.

[42] Bayer SA, Altman J, Paxinos G (2004). Developmental of the telencephalon: neural stem cells, neurogenesis, and neuronal migration.. The rat nervous system.

[43] Kennedy TE, Serafini T, de la Torre JR, Tessier-Lavigne M (1994). Netrins are diffusible chemotropic factors for commissural axons in the embryonic spinal cord.. Cell.

[44] Alvarez-Bolado G, Swanson L (1996).

[45] Briscoe J, Sussel L, Serup P, Hartigan-O'Connor D, Jessell TM (1999). Homeobox gene Nkx2.2 and specification of neuronal identity by graded Sonic hedgehog signalling.. Nature.

[46] Briscoe J, Ericson J (2001). Specification of neuronal fates in the ventral neural tube.. Curr Opin Neurobiol.

[47] Stuhmer T, Anderson SA, Ekker M, Rubenstein JL (2002). Ectopic expression of the Dlx genes induces glutamic acid decarboxylase and Dlx expression.. Development.

[48] Potter GB, Petryniak MA, Shevchenko E, McKinsey GL, Ekker M (2009). Generation of Cre-transgenic mice using Dlx1/Dlx2 enhancers and their characterization in GABAergic interneurons.. Mol Cell Neurosci.

[49] Elias CF, Lee CE, Kelly JF, Ahima RS, Kuhar M (2001). Characterization of CART neurons in the rat and human hypothalamus.. J Comp Neurol.

[50] Dallvechia-Adams S, Kuhar MJ, Smith Y (2002). Cocaine- and amphetamine-regulated transcript peptide projections in the ventral midbrain: colocalization with gamma-aminobutyric acid, melanin-concentrating hormone, dynorphin, and synaptic interactions with dopamine neurons.. J Comp Neurol.

[51] Elias CF, Sita LV, Zambon BK, Oliveira ER, Vasconcelos LA (2008). Melanin-concentrating hormone projections to areas involved in somatomotor responses.. J Chem Neuroanat.

[52] Leinninger GM, Jo YH, Leshan RL, Louis GW, Yang H (2009). Leptin acts via leptin receptor-expressing lateral hypothalamic neurons to modulate the mesolimbic dopamine system and suppress feeding.. Cell Metab.

[53] Arendt D, Nubler-Jung K (1999). Comparison of early nerve cord development in insects and vertebrates.. Development.

[54] Denes AS, Jekely G, Steinmetz PR, Raible F, Snyman H (2007). Molecular architecture of annelid nerve cord supports common origin of nervous system centralization in bilateria.. Cell.

[55] Arendt D, Denes AS, Jekely G, Tessmar-Raible K (2008). The evolution of nervous system centralization.. Philos Trans R Soc Lond B Biol Sci.

[56] Puelles L, Rubenstein JL (1993). Expression patterns of homeobox and other putative regulatory genes in the embryonic mouse forebrain suggest a neuromeric organization.. Trends Neurosci.

[57] Bagri A, Marin O, Plump AS, Mak J, Pleasure SJ (2002). Slit proteins prevent midline crossing and determine the dorsoventral position of major axonal pathways in the mammalian forebrain.. Neuron.

[58] Lin L, Isacson O (2006). Axonal growth regulation of fetal and embryonic stem cell-derived dopaminergic neurons by Netrin-1 and Slits.. Stem Cells.

[59] Hernandez-Montiel HL, Tamariz E, Sandoval-Minero MT, Varela-Echavarria A (2008). Semaphorins 3A, 3C, and 3F in mesencephalic dopaminergic axon pathfinding.. J Comp Neurol.

[60] Kolk SM, Gunput RA, Tran TS, van den Heuvel DM, Prasad AA (2009). Semaphorin 3F is a bifunctional guidance cue for dopaminergic axons and controls their fasciculation, channeling, rostral growth, and intracortical targeting.. J Neurosci.

[61] Yue Y, Widmer DA, Halladay AK, Cerretti DP, Wagner GC (1999). Specification of distinct dopaminergic neural pathways: roles of the Eph family receptor EphB1 and ligand ephrin-B2.. J Neurosci.

[62] Sieber BA, Kuzmin A, Canals JM, Danielsson A, Paratcha G (2004). Disruption of EphA/ephrin-a signaling in the nigrostriatal system reduces dopaminergic innervation and dissociates behavioral responses to amphetamine and cocaine.. Mol Cell Neurosci.

[63] Barresi MJF, Hutson LD, Chien C-B, Karlstrom RO (2005). Hedgehog regulated slit expression determines commissure and glial cell position in the zebrafish forebrain.. Development.

[64] Cooper MA, Kobayashi K, Zhou R (2009). Ephrin-A5 regulates the formation of the ascending midbrain dopaminergic pathways.. Dev Neurobiol.

[65] Stoykova A, Fritsch R, Walther C, Gruss P (1996). Forebrain patterning defects in Small eye mutant mice.. Development.

[66] Puelles L, Rubenstein JL (2003). Forebrain gene expression domains and the evolving prosomeric model.. Trends Neurosci.

[67] Deiner MS, Sretavan DW (1999). Altered midline axon pathways and ectopic neurons in the developing hypothalamus of netrin-1- and DCC-deficient mice.. J Neurosci.

[68] Xu C, Fan CM (2008). Expression of Robo/Slit and Semaphorin/Plexin/Neuropilin family members in the developing hypothalamic paraventricular and supraoptic nuclei.. Gene Expr Patterns.

[69] Cotta-Grand N, Rovere C, Guyon A, Cervantes A, Brau F (2009). Melanin-concentrating hormone induces neurite outgrowth in human neuroblastoma SH-SY5Y cells through p53 and MAPKinase signaling pathways.. Peptides.

[70] Schoofs L, Jegou S, Andersen AC, Tonon MC, Eberle AN (1988). Coexistence of melanin-concentrating hormone and alpha-melanocyte-stimulating hormone immunoreactivities in the central nervous system of the locust, Locusta migratoria.. Brain Res.

[71] St-Jacques B, Dassule HR, Karavanova I, Botchkarev VA, Li J (1998). Sonic hedgehog signaling is essential for hair development.. Curr Biol.

[72] Guyon A, Conductier G, Rovere C, Enfissi A, Nahon JL (2009). Melanin-concentrating hormone producing neurons: Activities and modulations.. Peptides.

[73] Guyon A, Nahon JL (2007). Multiple actions of the chemokine stromal cell-derived factor-1alpha on neuronal activity.. J Mol Endocrinol.

[74] Stanley S, Pinto S, Segal J, Perez CA, Viale A (2010). Identification of neuronal subpopulations that project from hypothalamus to both liver and adipose tissue polysynaptically.. Proc Natl Acad Sci U S A.

[75] Markakis EA, Swanson LW (1997). Spatiotemporal patterns of secretomotor neuron generation in the parvicellular neuroendocrine system.. Brain Res Brain Res Rev.

[76] Panganiban G, Rubenstein JL (2002). Developmental functions of the Distal-less/Dlx homeobox genes.. Development.

[77] Andrews W, Liapi A, Plachez C, Camurri L, Zhang J (2006). Robo1 regulates the development of major axon tracts and interneuron migration in the forebrain.. Development.

[78] Fellmann D, Bugnon C, Risold PY (1987). Unrelated peptide immunoreactivities coexist in neurons of the rat lateral dorsal hypothalamus: human growth hormone-releasing factor1-37-, salmon melanin-concentrating hormone- and alpha-melanotropin-like substances.. Neurosci Lett.

[79] Risold PY, Fellmann D, Rivier J, Vale W, Bugnon C (1992). Immunoreactivities for antisera to three putative neuropeptides of the rat melanin-concentrating hormone precursor are coexpressed in neurons of the rat lateral dorsal hypothalamus.. Neurosci Lett.

[80] Fellmann D, Bresson JL, Breton C, Bahjaoui M, Rouillon A (1989). Cloning of cDNAs encoding a rat neuropeptide immunologically related to salmon melanin-concentrating hormone.. Neurosci Lett.

[81] Bugnon C, Bahjaoui M, Fellmann D (1991). A simple method for coupling in situ hybridization and immunocytochemistry: application to the study of peptidergic neurons.. J Histochem Cytochem.

[82] Fernandez AS, Pieau C, Reperant J, Boncinelli E, Wassef M (1998). Expression of the Emx-1 and Dlx-1 homeobox genes define three molecularly distinct domains in the telencephalon of mouse, chick, turtle and frog embryos: implications for the evolution of telencephalic subdivisions in amniotes.. Development.

[83] Nieuwenhuis E, Barnfield PC, Makino S, Hui CC (2007). Epidermal hyperplasia and expansion of the interfollicular stem cell compartment in mutant mice with a C-terminal truncation of Patched1.. Dev Biol.

[84] Funato H, Saito-Nakazato Y, Takahashi H (2000). Axonal growth from the habenular nucleus along the neuromere boundary region of the diencephalon is regulated by semaphorin 3F and netrin-1.. Mol Cell Neurosci.

[85] Marillat V, Cases O, Nguyen-Ba-Charvet KT, Tessier-Lavigne M, Sotelo C (2002). Spatiotemporal expression patterns of slit and robo genes in the rat brain.. J Comp Neurol.

[86] Guijarro P, Simo S, Pascual M, Abasolo I, Del Rio JA (2006). Netrin1 exerts a chemorepulsive effect on migrating cerebellar interneurons in a Dcc-independent way.. Mol Cell Neurosci.

[87] Graham FL, Smiley J, Russell WC, Nairn R (1977). Characteristics of a human cell line transformed by DNA from human adenovirus type 5.. J Gen Virol.

[88] Pissios P, Ozcan U, Kokkotou E, Okada T, Liew CW (2007). Melanin concentrating hormone is a novel regulator of islet function and growth.. Diabetes.

